# Labor Supply, Risk Aversion, and Conflict Uncertainty

**DOI:** 10.1111/risa.70308

**Published:** 2026-07-14

**Authors:** Kiet Tuan Duong, Nam T. Vu

**Affiliations:** ^1^ University of York York UK; ^2^ Miami University Oxford Ohio USA

**Keywords:** conflict uncertainty, health, labor, safety and environmental (HS&E) risks

## Abstract

We argue that the presence of conflict uncertainty fundamentally re‐frames the labor‐leisure choice for individual agents. During normal times, supplying labor is often perceived as a safe choice. When working itself becomes perceived as a gamble due to conflict risk, an aversion to that specific risk will result in a measurable decrease in labor supply. Using proximity to the Ukrainian border as a plausibly exogenous proxy for conflict uncertainty vis‐à‐vis the Russian incursion into Ukraine in 2014, we show that an increase in conflict uncertainty negatively affects individuals' willingness to work. Moreover, this response also coincides with increased perceived risk aversion arising from the conflict.

## 1 Introduction

People perceive gains and losses asymmetrically, often leading to irrational choices under standard utility. Such a powerful idea, which is later canonically framed in the context of an *S*‐shaped utility function (Kahneman and Tversky 1979), implies a dichotomy in risk‐taking behavior: individuals are risk‐averse when choices are framed as gains, but then reverse course when the same choices are framed as losses. This line of argument and the introduction of prospect theory, a psychological theory of decision‐making under risk, have sparked a productive intellectual discussion of the interplay between perceived risk aversion (i.e., the attitudinal change caused by the activated loss aversion due to heightened risk) and the changes in individual choices it produces (i.e., revealed risk aversion).

Every single paper in this literature has significantly expanded our understanding of the nature of risks and, more importantly, how the perception of risks can impact various aspects of our lives, including but not limited to environmental (Ryan 2012), policy (Keeler 1994), and health (Currie et al. 2015) risks. Given Russia's 2014 invasion of Ukraine, another strand of the literature on geopolitical risks shows that conflicts have become an increasingly important source of risk and that geopolitical risks can have significant, tangible effects on the economy (Caldara and Iacoviello 2022; Caldara et al. 2026). Mostly, these strands of the literature have lived their separate lives. Moreover, despite this long intellectual history of research on risks and risk perception following Kahneman's seminal works (Kahneman and Tversky 1974;1979; Tversky and Kahneman 1981;1991), the extent to which uncertainty associated with conflicts affects individual choice by altering their perception of risk remains unclear.^1^

Our paper contributes to this discussion by examining how conflict‐related risks affect one's subjective (perceived) risk aversion due to the possibility of conflict‐related losses and, therefore, one's labor choices (i.e., revealed risk aversion). We argue that the presence of conflict uncertainty fundamentally re‐frames the labor‐leisure choice itself. During normal times, the decision to supply labor is often perceived as a “safe” choice. However, the introduction of conflict risk, even in the absence of direct physical risk, alters this perception. Working in this case is the risky option: the potential disruption to commuting, the psychological cost of being separated from family during a perceived crisis, and the economic uncertainty that the job or firm may not be stable. When working itself becomes the perceived gamble, an aversion to that specific risk will result in a measurable decrease in labor supply. To be clear, we explicitly consider a mechanism in which an exogenous shock (due to heightened conflict uncertainty) activates a specific psychological trait (i.e., loss aversion) via changes in *perceived risk aversion*. This, in turn, causes changes in behavior, which we refer to as *revealed risk aversion*.^2^

To fix intuition about how conflict risk affects labor choices, we build on Kahneman and Tversky (1979) and consider a stylized two‐period model in which the agent faces an *S*‐shaped utility function. The model builds on a growing body of evidence that reference‐dependent preferences shape labor supply decisions, as documented in the context of taxi drivers (Crawford and Meng 2011; Thakral and Tô 2021) and bicycle messengers (Fehr and Goette 2007). The agent has to decide how much labor ($L_{1}$) to supply in the first period, and the consequence of such a choice is realized in Period 2. As the primary driver of risk‐averse behavior is loss aversion in prospect theory, we introduce an effective loss function to capture the agent's *perceived risk aversion* (i.e., their effective loss from the conflict). With similar tokens, we use their observed labor supply choices to capture *revealed risk aversion*, as these choices reveal a higher aversion to the risk of working. The model suggests that higher conflict risk increases the agent's perceived risk aversion and decreases their optimal labor supply.

Based on these results, we examine two hypotheses. The first hypothesis concerns whether increased conflict uncertainty leads to changing labor supply choices (i.e., revealed risk aversion). The second hypothesis concerns whether changes in the individual's perceived risk aversion drive the documented changes in the labor supply response. Intuitively, conflicts often result in loss of life, environmental destruction, and decreased overall safety (Pereira et al. 2022; Lewtak et al. 2022).^3^ Therefore, our two hypotheses can also be interpreted as how increased health, safety, and environmental (HS&E) risks, driven by conflict uncertainty, influence individual labor decisions by increasing risk aversion.

To explore these hypotheses, we use proximity to the border with Ukraine as a plausibly exogenous proxy for exposure to conflict risks and examine how the increase in risks associated with negative externalities arising from the Russia–Ukraine conflict spillover into individual subjective risk aversion and how such spillover can affect their choices. Specifically, we leverage the European Social Survey (ESS), established in 1995 by the European Science Foundation and available biennially since 2002. For each participating country, the survey contains at least 1500 individuals, randomly selected to represent the country's population 15 and over. The scope of the data is impressive: since 2002, nearly 500,000 interviews have been completed across 39 countries.

Turning to the empirical approach, we examine how the 2014 conflict between Russia and Ukraine influenced the extent to which individuals residing in Poland, Hungary, and Slovakia (PHS) changed their labor supply choices and the degree of risk aversion. Residents of these neighboring countries have been uniquely positioned to experience the direct and indirect consequences of the war, including potential threats from being conflict victims (e.g., stray missiles). For example, on November 15, 2022, a missile strike in southeastern Poland killed two people, causing concerns about a broader escalation in the Russia‐Ukraine conflict. Investigations alleged that Ukrainian air defense systems likely fired the missile in response to a Russian missile attack.^4^ In addition to the missile incident, Poland faced increased security risks from Russian drone attacks.^5^ On the night of August 20–21, 2025, Russian forces launched a massive airstrike that targeted Mukachevo, a city in Ukraine's far west near the Hungarian border, with Kalibr missiles and drones, causing significant damage and civilian injuries.^6^ The attack, which included missile strikes on industrial sites and a drone crash in nearby Khust district, highlights the proximity of the conflict to Hungary, with local authorities responding to fires, injuries, and infrastructure damage in the Zakarpattia region.^7^ By considering individuals' perceptions in these countries, we aim to understand how geographic proximity to the conflict has shaped their perception of risks.

Our identification strategy uses variation in geographic proximity to the Ukrainian border as a proxy for the conflict's uncertainty. Intuitively, the closer an individual is to the border, the higher the probability that the conflict will directly impact such an individual. Examples of such impacts include, but are not limited to, reductions in overall health and safety and substantial deterioration of the living environment. Our identification strategy is motivated by the exogeneity associated with the conflict. In fact, the 2014 Russian incursion into Ukraine was largely unexpected, making it unlikely that PHS residents would relocate in anticipation of the event. Therefore, location choices are unlikely to be influenced by perceptions of the likelihood of incursion, and consequently, the conflict's later impact on risk perceptions is not driven by location.

Our benchmark empirical specifications leverage variation in exposure to conflict risks for individuals near the border with Ukraine, relative to individuals residing outside such a region, in a difference‐in‐differences framework. Our dependent variables of interest are whether the survey respondent has worked in paid employment in the last seven days and whether there is a recent increase in the need to avoid unsafe areas (i.e., perceived risk aversion). By including various individual characteristics, we control for intrinsic risk aversion and focus on how perceived risks associated with conflict uncertainty (proximity to the conflict) affect labor choices via revealed risk aversion. Indeed, our approach is motivated by the large literature on the subject, showing how risk aversion is impacted by gender (Jianakoplos and Bernasek 1998; Schubert et al. 1999; Borghans et al. 2009), age (Bellante and Green 2004; Hanoch et al. 2018), employment status (Chetty and Szeidl 2007; Backus and Esteller‐Moré 2017), the time the individual typically spends on media (Allcott et al. 2020), marital status (Roussanov and Savor 2014), education (Jung 2015), and citizenship (Woodland and Yoshida 2006), among others.

We document the following two results. First, individuals exposed to higher conflict uncertainty are more likely to reduce their labor supply in response to the Russia‐Ukraine conflict than nonexposed individuals. Second, increased risk aversion due to heightened conflict uncertainty can explain negative labor supply responses. These results are consistent across a battery of robustness checks. First, we consider an alternative measure of labor supply choice. Second, we conduct two placebo tests, in which we randomize the individuals' locations and the timing of the conflicts. Third, we consider several alternative clustering methods for the standard errors. Fourth, we consider a variety of additional controls that capture individuals' perceptions of immigration, residence tenure, and other environmental characteristics.

Indeed, models incorporating a precautionary savings motive (see, e.g., Leland 1968; Sandmo 1970; Lundberg 1985; Kimball 1990) typically imply that an increase in uncertainty should lead individuals to increase their current labor supply as they build up their savings to self‐insure against potential future negative shocks. Our quasi‐natural experiment creates a clear tension: does the “economic” agent work more, or does the “behavioral” agent work less? We contribute to this literature by showing that when the source of uncertainty is a salient physical threat (HS&E risk), the behavioral response dominates.^8^ Our central contribution is to posit and test a psychological spillover effect: we argue that the HS&E risk is the trigger that activates the loss‐aversion channel (à la Kahneman and Tversky 1979), which we then capture empirically via its proxy, “perceived risk aversion,” to explain the negative labor supply response.

Our paper is also related to the growing literature examining how external shocks alter risk preferences (see, e.g., Camerer et al. 1997; Hanaoka et al. 2018; Aragón et al. 2020; Akesaka et al. 2023). While such literature examines how realized shocks impact risk aversion, we find that conflict uncertainty, unresolved threat of conflict that heightens the salience of potential losses, increases it. In addition, we examine the interplay between perceived and revealed risk aversion through labor supply responses, providing direct evidence that attitudinal shifts translate into behavioral changes.

Our paper also contributes to a growing literature documenting the mixed effects of conflict on risk aversion (see, e.g., Callen et al. 2014; Voors et al. 2012). Specifically, Callen et al. (2014) find that exposure to violence and fear can lead to increases in risk aversion, which dovetails nicely with our theoretical exposition. In stark contrast, Voors et al. (2012) document that exposure to conflict can make people *more* risk‐seeking. Along similar lines, Bauer et al. (2016) document that conflict exposure can shift social and risk preferences in ways that persist well beyond the cessation of violence. Our paper thus contributes to this discussion by providing evidence that conflict uncertainty drives risk aversion, whereas *realized* violence may have different implications.

Our paper proceeds as follows: Section 2 describes the theoretical foundation and the two hypotheses considered. Section 3 describes our identification strategy and the dataset used in the empirical analyses. Section 4 presents the empirical results, in which we examine the validity of the two hypotheses considered in Section 2. We conclude in Section 5.

## 2 Theoretical Predictions and Hypotheses

### 2.1 Model

To formalize the extent to which conflict risk affects labor choices, we build on Kahneman and Tversky (1979) and consider a two‐period model in which the subscript t denotes time t=1,2. An agent has to decide how much labor ($L_{1}$) to supply in the first period, and the consequence of such a choice is realized in period 2.^9^

#### 2.1.1 Preferences

Motivated by Kahneman and Tversky (1979), Tversky and Kahneman (1992), Kőszegi and Rabin (2006), and Kőszegi and Rabin (2007), we define the agent's utility function over gains and losses relative to a reference point R. Specifically, prospect theory value function v(·) writes:

(1) $$\begin{array}{c} v\left(x-R\right)=\left\{\begin{array}{cc} {\left(x-R\right)}^{\alpha } & \text{if}\;x\geq R\;\text{(Gains)}\\ -\lambda {\left(R-x\right)}^{\beta } & \text{if}\;x<R\;\text{(Losses)} \end{array}\right. \end{array}$$

where R is the reference point (e.g., subsistence consumption, or “normal” life), λ>1 is the agent's constant, baseline coefficient of loss aversion, and α∈(0,1) and β∈(0,1) capture diminishing sensitivity. For simplicity, we assume α=β.^10^ In addition to the prospect theory value function (1), the agent also derives standard utility from leisure, $u(1-L_{1})$, which we assume to have a canonical Constant Relative Risk Aversion (CRRA) functional form:

(2) $$\begin{array}{c} u\left(1-L_{1}\right)=\frac{{\left(1-L_{1}\right)}^{1-\sigma }}{1-\sigma }, \end{array}$$

where σ>0 represents the degree of relative risk aversion.^11^

We assume away Arrow–Debreu securities; that is, a complete market for state‐contingent claims against conflict risk does not exist. In the context of the Ukrainian conflict, the lack of such securities implies that the risk of loss due to conflict is uninsurable, and the *only* margin of adjustment against such risk is for agents to change their own behavior (Ehrlich and Becker 1972).

#### 2.1.2 Payoffs

In Period 1, the agent chooses labor $L_{1}\in \left[0,1\right]$ to maximize their total discounted utility, while earning a wage of $w_{1}$. We assume no savings for simplicity. Without the ability to save, consumption in Period 1 is $C_{1}=w_{1}L_{1}$. Total utility of the agent in Period 1 is therefore^12^

(3) $$\begin{array}{c} U_{1}=v\left(C_{1}-R_{1}\right)+u\left(1-L_{1}\right)={\left(w_{1}L_{1}-R_{1}\right)}^{\alpha }+\frac{{\left(1-L_{1}\right)}^{1-\sigma }}{1-\sigma }. \end{array}$$

In the second period, the agent is exposed to conflict risk, and the risk generated by their labor choice in the first period is realized. Since working during conflict is inherently riskier, we assume that supplying labor increases exposure to conflict risk. To maintain tractability, we assume that the probability of a bad state $q(L_{1})$ is an increasing function of labor supplied in period 1 ($q^{\prime }\left(L_{1}\right)>0$).

To capture the notion that the HS&E risk from the conflict (parameterized by θ≥1) acts as a psychological trigger that activates and intensifies loss aversion, we allow the loss aversion parameter itself to be a state‐dependent function of the conflict risk. We define the agent's *effective loss aversion* as $\lambda_{E}\equiv f\left(\theta \right)$, where f(θ)≥λ>1 and $f^{\prime }\left(\theta \right)>0$. Motivated by De Giorgi and Post (2011), who use a state‐dependent reference point, this assumption implies that the conflict renders the agent more psychologically sensitive to all losses. The utility of the catastrophic loss K is thus $v\left(-K\right)=-f\left(\theta \right){\left(K\right)}^{\beta }$. Our assumption of the agent's effective loss aversion is motivated by the related literature on how fear can activate loss aversion (see, e.g., Loewenstein 2000; Caplin and Leahy 2001). Specifically, a common theme in such literature is that “visceral factors” such as fear and anxiety can alter deep parameters that specify agents' preferences.^13^

In the good state, which happens with a probability of $1-q(L_{1})$, the agent survives unharmed with an outcome of $R_{2}$. Their utility in this case is $v\left(R_{2}-R_{2}\right)=v\left(0\right)=0$. In the bad state, which happens with a probability of $q(L_{1})$, the agent suffers a catastrophic outcome with a loss of K relative to the reference point. The agent's expected utility for Period 2, discounted by δ∈(0,1], is thus

(4) $$\begin{array}{ccc} E[U_{2}] & = & \delta \left[\left(1-q\left(L_{1}\right)\right)\cdot v\left(0\right)+q\left(L_{1}\right)\cdot v\left(-K\right)\right]\\ & = & \delta \left[-q\left(L_{1}\right)f\left(\theta \right){\left(K\right)}^{\beta }\right] \end{array}$$

Taken together, the agent chooses $L_{1}$ to maximize their total lifetime utility, $U=U_{1}+E\left[U_{2}\right]$:

(5) $$\begin{array}{c} \max_{L_{1}}\;U=\left[{\left(w_{1}L_{1}-R_{1}\right)}^{\alpha }+\frac{{\left(1-L_{1}\right)}^{1-\sigma }}{1-\sigma }\right]-\delta f\left(\theta \right){\left(K\right)}^{\beta }q\left(L_{1}\right), \end{array}$$

where the first‐order condition (FOC) with respect to $L_{1}$, $\frac{\partial U}{\partial L_{1}}=0$, writes

(6) $$\begin{array}{c} \underset{\text{MB of Labor (Gain in}\;C_{1})}{\underset{︸}{\alpha w_{1}{\left(w_{1}L_{1}-R_{1}\right)}^{\alpha -1}}}-\underset{\text{MC of Labor (Loss of Leisure)}}{\underset{︸}{{\left(1-L_{1}\right)}^{-\sigma }}}-\underset{\text{MC of Labor (Future Loss)}}{\underset{︸}{\delta f\left(\theta \right){\left(K\right)}^{\beta }q^{\prime }\left(L_{1}\right)}}=0, \end{array}$$

where the first term represents the marginal benefits (MB) of labor, and the last two terms represent the marginal costs (MC).^14^

#### 2.1.3 Model Implications: Perceived Versus Revealed Risk Aversion

Our model links the unobserved conflict risk θ to two observable outcomes. First, it increases the agent's unobserved *effective loss aversion*, f(θ), which (by Remark 1) causes a change in their chosen behavior, $L_{1}^{*}$ (our measure of *revealed risk aversion*). Second, it also causes a general psychological shift, as measured by attitudinal survey questions.

Remark 1 An increase in the conflict risk parameter (θ) leads to:

1. An increase in the agent's perceived risk aversion ($\lambda_{E}\equiv f\left(\theta \right)$).
2. A decrease in the agent's optimal labor supply $L_{1}^{*}$ (an increase in revealed risk aversion).

The first part of Remark 1 follows from the assumption regarding the monotonicity of f(θ). Our second result from Remark 1 arises from the first order condition with respect to $L_{1}$. Intuitively, since θ only appears on the MC side, an increase in θ increases f(θ), which increases the MC of working for any given $L_{1}$. Since MB is unchanged with changes in θ, the agent must reduce $L_{1}$ in equilibrium. We leave the detailed derivations of Remark 1 in the Supporting Information for brevity. The appendix in the Supporting Information also shows the analogous result holds in the loss domain.

**FIGURE 1:**
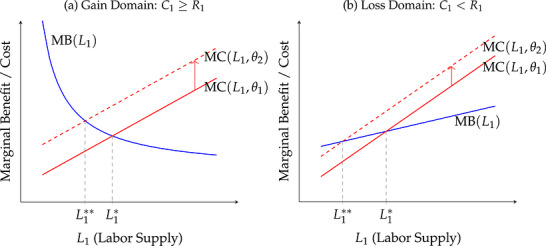
Effect of an increase in conflict risk (θ).

To visualize the mechanism behind Remark 1, we plot the marginal benefit and marginal cost for the agent under two regions of its utility function (gains and losses) in Figure 1. An increase in conflict risk (from $\theta_{1}$ to $\theta_{2}$) shifts the MC curve up, and results in a lower optimal labor choice $L_{1}^{**}$, regardless of whether the agent is subject to the gain or loss region.

### 2.2 Testable Hypotheses

The model provides two implications. The first implication has to do with the extent to which exposure to uncertainty arising from conflict will negatively impact individual *overall* perception with respect to their safety. Intuitively, given the increased exposure to risks associated with the war, individuals become perceptively more risk‐averse. The second implication is that such changes in risk perception lead individuals to alter the types of activities they aspire to engage in and the extent to which they value various aspects of their lives.

Hypothesis I (Revealed Risk Aversion) Increased uncertainty associated with conflicts reduces an individual's labor supply.
Hypothesis II (Perceived Risk Aversion) Such a fall in labor supply coincides with an increase in the individual's perceived risk aversion.

Since conflicts and their aftermaths may involve loss of life, destruction of the living environment, and an overall decrease in safety, increased conflict uncertainty may lead to increased HS&E risks. As a result, another interpretation of these hypotheses is that the extent to which an increase in HS&E risks (Pereira et al. 2022; Lewtak et al. 2022), as captured by the rise in conflict uncertainty, impacts individual labor choice via a corresponding rise in risk aversion. Indeed, the interplay between risk aversion and a psychological theory of decision‐making under risk has sparked a productive intellectual discussion on the relationship between risk and the emotional responses it elicits (Slovic et al. 2004), risk perception (Siegrist and Gutscher 2006), cognitive biases (Posner 1998), among others.

## 3 Empirical Approach

This section outlines our empirical approach to test the two hypotheses considered in Section 2. In particular, we discuss our identification strategy and the empirical specifications used to examine the validity of the two hypotheses in Section 2.

### 3.1 Identification Strategy and Empirical Specifications

We examine how the ongoing conflict between Russia and Ukraine has influenced the perceived and revealed risk aversion for individuals residing in Poland, Hungary, and Slovakia (PHS). Although Romania borders Ukraine, the European Social Survey (ESS) does not provide post‐2014 data for Romania. As a result, our analysis cannot observe the impact of the Russia–Ukraine conflict on the Romanian individuals. We therefore exclude Romania from our sample. Given their unique proximity to Ukraine, PHS provides us with a natural experiment to study whether uncertainty, as a result of the war, led to changes in their citizens' perceptions related to health and safety. Intuitively, people living in areas that share borders with Ukraine are more exposed to the HS&E risks (e.g., being a war casualty due to stray missiles, immigrants' diseases, and air quality) than others who live further away. Thus, the former are more exposed to heightened conflict risks. By leveraging variation in geographic proximity to the Ukrainian border across regions within these countries, we aim to understand how conflict uncertainty has shaped individuals' perceived and revealed risk aversion.

Our identification strategy thus hinges on using proximity to the Ukrainian border as a proxy for exposure to the *perceived* risks posed by stray missiles from the conflict and potential clashes between Russia and Ukraine. We note that our focus is to capture the effects of the *perceived* risks associated with such a conflict (i.e., the possibility of a stray missile or other negative externalities associated with the war), not on the real (i.e., actual realization) of any destruction associated with it.

As the Russian invasion of Ukraine in 2014 was largely unexpected for the general public, it is unlikely to prompt residents of PHS to relocate in anticipation of the event. Thus, whether one's location is at or near the border is unlikely to depend on one's perception of the likelihood of the 2014 Russian incursion. In other words, PHS residents' location choices relative to the border are unlikely to determine how the conflict later influences their risk perceptions.

Russia's 2014 incursion into Ukraine provides a plausibly exogenous shock to conflict uncertainty for residents of neighboring countries. The incursion began in late February 2014 with the seizure of Crimea and escalated by April to armed conflict in eastern Ukraine. For our purposes, two features of this event are central. First, the conflict was largely unexpected. Second, it introduced tangible risks to border residents: the possibility of stray missiles and the resulting deterioration of infrastructure and economic conditions. Poland alone shares over 500 km of border with Ukraine. Intuitively, residents in PHS border regions faced heightened exposure to HS&E risks compared to their counterparts in interior regions. Our identification strategy hinges on this geographic variation in exposure to conflict uncertainty.

Turning to more details of our identification strategy, we classify the regions within PHS into two main categories based on the Nomenclature of Territorial Units for Statistics (NUTS) code. The first category includes regions near the Ukrainian border, while the second includes regions not near the Ukrainian border. Naturally, this geographical dichotomy allows us to isolate survey respondents into two groups. The first group consists of respondents living in regions bordering Ukraine, and the second group consists of respondents living outside such regions. To visualize the locations of these two groups and their corresponding regions relative to PHS as a whole, we present the regions along the border with Ukraine in Figure 3.

**FIGURE 2:**
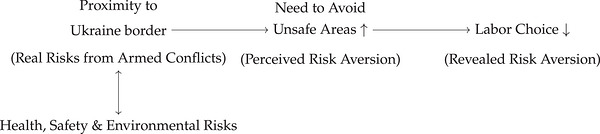
Conflict uncertainty, risk aversion, and labor supply. *Note*: This diagram shows how conflict uncertainty can impact individual labor choices via increased risk aversion. Given the destructive nature of conflicts, it also shows the interplay between conflict uncertainty and health, safety, & environmental (HS&E) risks.

**FIGURE 3:**
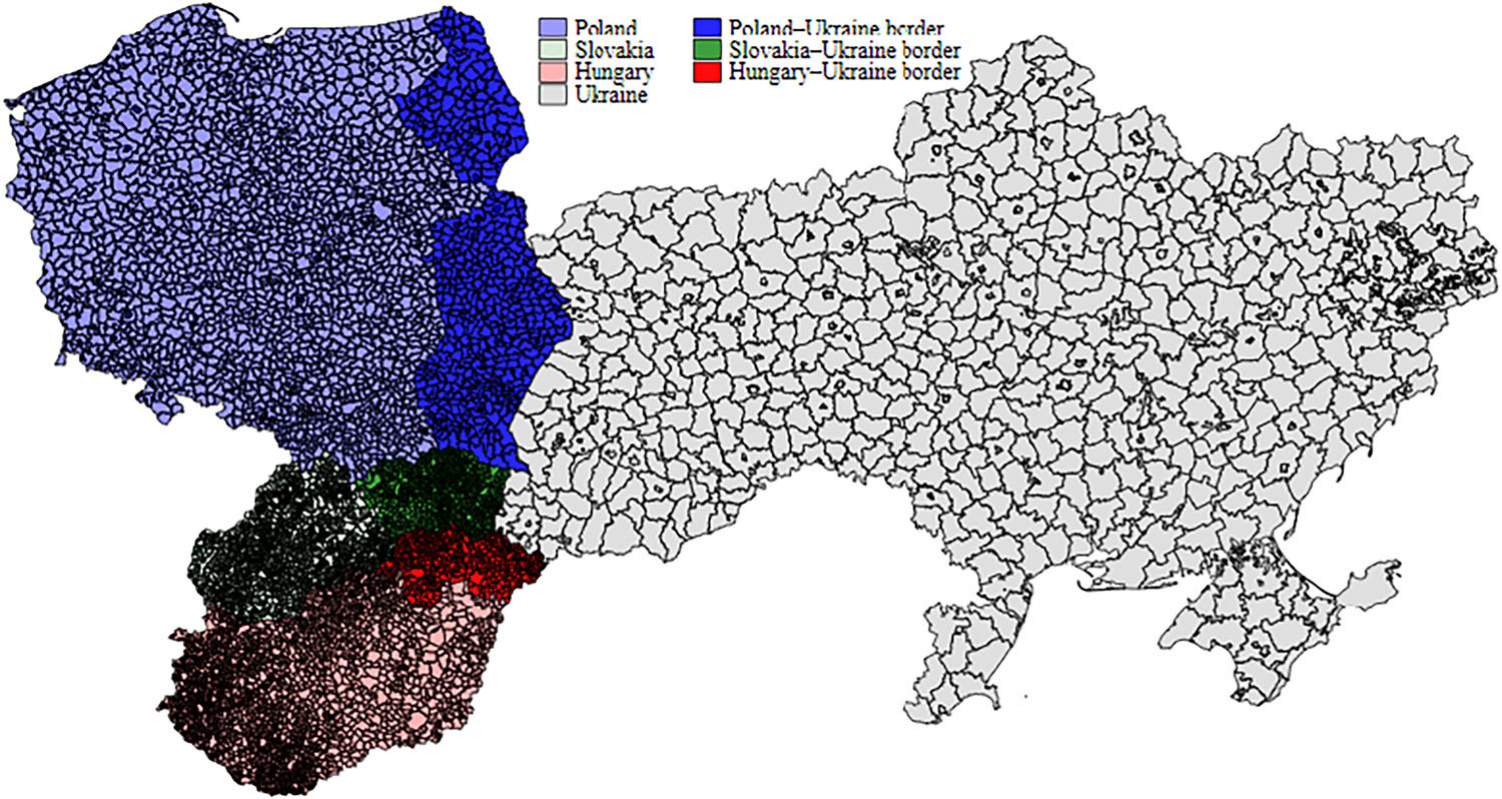
Regions sharing borders with Ukraine. *Note*: This figure presents a map of four countries: Poland, Hungary, Slovakia, and Ukraine. Poland is shown in blue, with the regions bordering Ukraine (i.e., Lubelskie, Podkarpackie, and Podlaskie) highlighted in dark blue. Hungary is shown in red, with the regions bordering Ukraine (i.e., Borsod‐Abaúj‐Zemplén and Szabolcs‐Szatmár‐Bereg) highlighted in dark red. Slovakia is shown in green, with the regions bordering Ukraine (i.e., Košický kraj and Prešovský kraj) highlighted in dark green. Ukraine is shown in gray.

Figure 3 shows the Polish, Hungarian, and Slovakian regions bordering Ukraine. We use regional information from the European Social Survey (ESS) to identify areas bordering Ukraine. However, there are differences in the use of regional classifications across the ESS waves. As the pre‐2010 ESS waves primarily use regional names, we manually check the regional names with NUTS (Nomenclature of Territorial Units for Statistics) Level 2 (NUTS‐2) and Level 3 (NUTS‐3) regions, as shown in Panel A of our Table A2 in the Supporting Information. After 2010, as shown in Panel B of our Table A2 in the Supporting Information, the ESS uses a mix of NUTS‐2 and NUTS‐3 classifications. Specifically, NUTS‐2 regions are used for Poland, whereas NUTS‐3 regions are used for Hungary and Slovakia. As presented in Table A2 in the Supporting Information, the first region is Lubelskie, located in southeastern Poland and bordering Ukraine's Volyn Oblast. The second region is Podkarpackie, in southern Poland, which borders Ukraine's Lviv Oblast. The third region is Podlaskie Voivodeship in northeastern Poland. While Podlaskie primarily borders Belarus and Lithuania rather than Ukraine directly, it is a historic borderland where Polish, Belarusian, and Ukrainian cultures intersect, with the Bug River often serving as a cultural and historical boundary. The Hungarian regions bordering Ukraine include Borsod‐Abaúj‐Zemplén and Szabolcs‐Szatmár‐Bereg. Before 2010, we relied on regional names, such as North Region and North Plain for Hungary, to identify areas bordering Ukraine, rather than on NUTS‐3 codes. Finally, in Slovakia, Košický kraj and Prešovský kraj are the regions neighboring Ukraine. Therefore, survey respondents residing in the dark‐shaded regions belong to the first group, while those residing outside such regions belong to the second group.

In the following sections, we are going to describe how we leverage this identification strategy in understanding the extent to which conflict uncertainty induces changes in both revealed (i.e., via the survey respondents' individual choices) and perceived risk aversion (i.e., via how these respondents reported changing their perceived need to avoid unsafe areas). We then show how the two are tightly linked: individuals reporting increased *perceived* risk aversion are also those who exhibit *revealed* risk aversion by working fewer hours beyond those attributable to their employers, health conditions, financial well‐being, and other demographic and economic factors.

#### 3.1.1 Uncertainty and Labor Choice

To disentangle how uncertainty arising from the Russia‐Ukraine conflict impacts residents' labor choice via revealed risk aversion (i.e., **Hypothesis I**), we consider the following regression specification

(7) $$\begin{array}{ccc} \text{Working}\;{\text{Choice}}_{i,t} & = & \alpha +\beta_{1}\text{Border with}\;{\text{Ukraine}}_{i}\times \text{Post}\;{\text{2014}}_{t}\\ & & +\;\;\beta_{2}\text{Border with}\;{\text{Ukraine}}_{i}+\beta_{3}\text{Post}\;{\text{2014}}_{t}\\ & & +\;\;\zeta X_{i,t}+\lambda_{c}+\varphi_{s}+\delta_{o}+\gamma_{e}+\varepsilon_{i,t}, \end{array}$$

where $\text{Working}\;{\text{Choice}}_{i,t}$ is a binary variable that captures whether resident i in time t has been working for the last seven days (i.e., employee, self‐employed, working for your family business) at the time of the survey, and $\text{Post}\;{\text{2014}}_{t}$ is an indicator function that takes a value of one if the observation is in or after 2014 and zero otherwise. $\text{Border with}\;{\text{Ukraine}}_{i}$ is a binary variable that indicates whether individual i resides in a locality that shares a border with Ukraine as illustrated in Figure 3. Our coefficient of interest is $\beta_{1}$, which captures the probability that an individual living in localities that share a border with Ukraine works relative to that of an individual residing in localities *not* sharing a border with Ukraine. $\lambda_{c}$, $\varphi_{s}$, $\delta_{o}$, and $\gamma_{e}$ are the country, income source, occupation, and highest education level fixed effects, respectively. We estimate Equation (7) using robust standard errors and report the marginal effects at the means throughout the paper.

In Equation (7), $X_{i,t}$ denotes the set of control variables. Our choice of the variables to include in $X_{i,t}$ follows the large literature on risk aversion, showing how it is impacted by gender (Jianakoplos and Bernasek 1998; Schubert et al. 1999; Borghans et al. 2009), age (Bellante and Green 2004; Hanoch et al. 2018), employment status (Chetty and Szeidl 2007; Backus and Esteller‐Moré 2017), the time the individual typically spends on media (Allcott et al. 2020), marital status (Roussanov and Savor 2014), education (Jung 2015), political identity (Jeleva and Rossignol 2009; Cai et al. 2020), and citizenship (Woodland and Yoshida 2006), among others.

We first control for infrastructural and individual demographic characteristics. Interrupted energy supply is a binary variable that equals 1 if there are concerns that energy supplies could be interrupted by technical failures or terrorist attacks, and 0 otherwise.^15^ Female gender is a binary variable that equals one if the individual is female and zero if the individual is male. Our inclusion of the individual's gender‐binary variable in the control group is motivated by related literature indicating that gender can significantly affect individual risk aversion (Jianakoplos and Bernasek 1998; Schubert et al. 1999; Borghans et al. 2009). Following the literature connecting age to individual risk aversion (Bellante and Green 2004; Hanoch et al. 2018), our set of controls includes age, which captures the individual's age. Marital Status is a binary variable that equals one if the individual is married (i.e., legally married or in a legally registered civil union), and zero otherwise (Roussanov and Savor 2014). Born in the Same Country is a binary variable indicating whether the individual was born in the country in which they were interviewed. Citizen of the Country is a binary variable indicating whether the individual holds citizenship in the country being interviewed (Woodland and Yoshida 2006).

Given that an individual might decide not to work simply due to their unemployment status and that their employment status also impacts their perceived risk aversion (Feinberg 1977; Chetty and Szeidl 2007; Backus and Esteller‐Moré 2017), we control for Unemployed 3 Months Plus, a binary variable equal to one if the individual experienced being unemployed for a period more than 3 months and zero otherwise. Given the documented bias in risk aversion concerning the time spent on media documented in the literature (Baron 2006; Allcott et al. 2020), our control variables include time spent on media, which is a binary variable that captures whether the individual has spent more than 2.5 h on an average weekday. Children at Home is a binary variable indicating whether the individual has any children living at home (Roussanov and Savor 2014). Knowledge/skills training is a binary variable that equals one if the individual has attended courses/conferences to improve knowledge and skills in the last 12 months, and zero otherwise (Jung 2015). Another reason why an individual might not work is their health. Thus, we include permanently sick, a binary variable that equals one if the individual is permanently sick or disabled, and zero otherwise. Second, it is also possible that an individual's labor choice can be impacted by their perception and satisfaction toward the country's state of economy, state of health services, and state of education (Chetty and Szeidl 2007). All these ‘state’ variables are on a scale of 0–10, where 0 represents the lowest satisfaction and 10 the highest. Finally, we control the individual's trust in political parties, trust in country's parliament, trust in politicians, and trust in the police (Jeleva and Rossignol 2009; Cai et al. 2020). Similarly, these “trust” variables range from 0 to 10, with 0 indicating the lowest trust and 10 the highest.

While it remains a possibility that certain contemporaneous developments could have biased our estimates in Equation (7), two features of the 2014 setting work in our favor. First, the Russian incursion was an unexpected and localized shock, as it is difficult to identify an alternative event in 2014 that would have differentially affected the border regions compared with other non‐border regions. Second, broad macroeconomic changes would have affected all regions within a country and, therefore, are absorbed by the country fixed effects in our specifications.^16^

#### 3.1.2 Mechanism: Increased Risk Aversion

Following the extensive and influential literature on the effects of risk perception on individual behavior (Kahneman and Tversky 1979; Kahneman 2011), we examine how risk perception affects an individual's labor choice. In particular, we posit that individuals facing uncertainty arising from the Russia–Ukraine conflict are more inclined to reduce their working hours due to a change in their subjective risk aversion. Figure 2 illustrates this mechanism, in which heightened uncertainty due to proximity to the conflict can change an individual's subjective risk aversion, which can impact their choice of labor supply.

To disentangle how conflict proximity causes changes in labor choice via altering an individual's (subjective) perceived risk aversion (i.e., **Hypothesis II**), we first consider whether proximity to the conflict can change an individual's subjective aversion to risks. Specifically, we estimate the following regression specification

(8) $$\begin{array}{ccc} \text{Need to Avoid Unsafe}\;{\text{Areas}}_{i,t} & = & \alpha +\beta_{1}\text{Border with}\;{\text{Ukraine}}_{i}\times \text{Post}\;{\text{2014}}_{t}\\ & & +\;\beta_{2}\text{Border with}\;{\text{Ukraine}}_{i}+\beta_{3}\text{Post}\;{\text{2014}}_{t}\\ & & +\;\zeta X_{i,t}+\lambda_{c}+\varphi_{s}+\delta_{o}+\gamma_{e}+\varepsilon_{i,t}, \end{array}$$

where $\text{Need to Avoid Unsafe}\;{\text{Areas}}_{i,t}$ is a binary variable that captures whether resident i at time t reported having observed increases in the need to avoid unsafe areas (i.e., perceived risk aversion). $\text{Border with}\;{\text{Ukraine}}_{i}$ is a dummy variable that indicates whether individual i resides in a locality that shares border with Ukraine, and $\text{Post}\;{\text{2014}}_{t}$ is an indicator function that takes a value of one if the observation is in or after 2014 and zero otherwise. $X_{i,t}$ denotes the same set of control variables defined in Equation (7). $\lambda_{c}$, $\varphi_{s}$, $\delta_{o}$, and $\gamma_{e}$ are the country, income source, occupation, and highest education level fixed effects, respectively. The specification employs robust standard errors. $\beta_{1}$ captures the extent to which the perception of health and safety risks for an individual living in localities that share a border with Ukraine is relative to that of an individual living in localities *not* sharing a border with Ukraine.

Having confirmed that proximity to the conflict can alter an individual's subjective aversion to risk, we next re‐estimate Equation (7) for two groups. The first group includes individuals who reported an increase in their subjective risk aversion. The second group includes individuals who reported no increase in their subjective risk aversion. **Hypothesis II** suggests that we should expect much of the decrease in labor supply to be driven by the former group but not necessarily by the latter group.

### 3.2 Data

Our primary data source is the ESS,^17^ initially developed in 1995 by the European Science Foundation. This project aims to establish a reliable dataset to support academics, researchers, and policymakers in analyzing and comparing social attitudes, beliefs, and behaviors across European countries. Since 2002, the project has conducted face‐to‐face interviews every two years, targeting at least 1500 individuals in each participating country, selected through rigorous random sampling from population, household, or address‐based sampling frames. These individuals represent the entire residential population aged 15 and over in each country. Almost 500,000 interviews have been conducted in 39 countries since 2002.

Our identification strategy is to study neighboring countries of Ukraine and empirically examine how people residing in regions bordering Ukraine perceive the risks inherent in the 2014 Russian–Ukrainian conflict. Therefore, we focus on Poland, Hungary, and Slovakia from the ESS data. Specifically, our sample covers 11 survey waves^18^ conducted in 2002, 2004, 2006, 2008, 2010, 2012, 2014, 2016, 2018, 2020, and 2023.^19^

In Panel A of Table 1, we present the sample distribution across the three countries and years. Among them, Poland accounts for the lion's share of our sample at 37.73%.^20^ Panel B presents the descriptive statistics for our variables of interest. Overall, there are 50,440 unique respondents from Poland, Hungary, and Slovakia across the eleven waves.

**TABLE 1:** Sample distribution and descriptive statistics.

Panel A: Sample distribution across countries and years								
	Full sample		Poland		Hungary		Slovakia	
	Obs.	%	Obs.	%	Obs.	%	Obs.	%
2002	3795	7.524	2110	11.088	1685	8.997		
2004	3228	6.400	1716	9.018			1512	11.921
2005	1498	2.970			1498	7.999		
2006	3967	7.865	1719	9.034	1364	7.283	884	6.970
2007	1036	2.054	2	0.011	154	0.822	880	6.938
2008	2580	5.115	1449	7.615			1131	8.917
2009	2393	4.744	170	0.893	1544	8.244	679	5.354
2010	4598	9.116	1713	9.002	1560	8.330	1325	10.447
2011	559	1.108	33	0.173			526	4.147
2012	5178	10.266	1879	9.874	1892	10.103	1407	11.094
2013	563	1.116	13	0.068	119	0.635	431	3.398
2015	3307	6.556	1614	8.482	1693	9.040		
2016	1379	2.734	1379	7.247				
2017	1919	3.805	310	1.629	1609	8.591		
2018	977	1.937	977	5.134				
2019	3250	6.443	513	2.696	1656	8.842	1081	8.523
2020	1994	3.953	1994	10.479				
2021	3235	6.414			1839	9.820	1396	11.007
2023	4633	9.185	1087	5.712	2115	11.293	1431	11.283
2024	351	0.696	351	1.845				
Total	50,440	100.000	19,029	100.000	18,728	100.000	12,683	100.000

Panel B: Descriptive statistics						
	Obs.	Mean	Std.	Median	Min	Max
Working Choice	50,440	0.502	0.500	1.000	0.000	1.000
Working Hours	41,714	53.374	80.070	40.000	0.000	555.000
Working Hours (log)	41,714	3.759	0.513	3.714	0.000	6.321
Need to Avoid Unsafe Areas	47,325	0.920	0.272	1.000	0.000	1.000
Border with Ukraine	50,440	0.201	0.401	0.000	0.000	1.000
Post 2014	50,440	0.417	0.493	0.000	0.000	1.000
Interrupted Energy Supply	50,440	0.954	0.210	1.000	0.000	1.000
Female Gender	50,360	0.551	0.497	1.000	0.000	1.000
Age	50,120	47.812	18.511	48.000	14.000	97.000
Marital Status	50,440	0.312	0.463	0.000	0.000	1.000
Born in the Same Country	50,398	0.983	0.128	1.000	0.000	1.000
Citizen of the Country	50,396	0.996	0.065	1.000	0.000	1.000
Unemployed 3 Months Plus	50,440	0.267	0.443	0.000	0.000	1.000
Time Spent on Media	50,440	0.793	0.405	1.000	0.000	1.000
Children at Home	50,270	0.416	0.493	0.000	0.000	1.000
Knowledge/Skills Training	50,440	0.187	0.390	0.000	0.000	1.000
Permanently Sick	50,440	0.029	0.168	0.000	0.000	1.000
State of Economy	48,756	3.983	2.358	4.000	0.000	10.000
State of Health Services	49,106	3.785	2.471	4.000	0.000	10.000
State of Education	46,131	5.041	2.402	5.000	0.000	10.000
Trust in Political Parties	45,403	2.932	2.357	3.000	0.000	10.000
Trust in Country's Parliament	49,130	3.626	2.560	3.000	0.000	10.000
Trust in Politicians	49,276	2.943	2.384	3.000	0.000	10.000
Trust in the Police	49,563	5.202	2.599	5.000	0.000	10.000

*Note*: This table presents the sample distribution across the countries that share borders with Ukraine (i.e., Poland, Hungary, and Slovakia) across years in Panel A. Because our research focuses on Russia's 2014 invasion of Ukraine, we only use the sample of countries sharing borders with Ukraine in our baseline regressions. The descriptive statistics for our variables of interest in the baseline regressions. Therefore, we also report working hours in this table after excluding these cases. Table A1 in the Supporting Information provides more details on the variable definitions and construction. In the Tobit specification, we censor $\text{Working Hours}\;{\text{(log)}}_{\mathrm{it}}$ at an upper bound of 5.13, corresponding to the log of 1 plus the maximum feasible number of hours per week.

We present descriptive statistics in Panel B of Table 1. On average, individuals in the sample are 48 years old, female, unmarried, born in the countries where they were interviewed, and citizens of those countries. In our sample, 26.7% of individuals experienced unemployment in the last 3 months, 79.3% spent more than 2.5 h on media per week, 41.6% had children at home, 18.7% attended courses to improve knowledge and skills in the last 12 months, and 2.9% were permanently sick or disabled. In terms of individuals' satisfaction with the economy, health services, and education, the majority are satisfied with the education system but not with the economy or health services. Most individuals do not trust political parties, the country's parliament, or politicians, but trust the police. The mean differences between the treated group (i.e., individuals in regions bordering Ukraine) and the control group (i.e., the ones without bordering Ukraine) are presented in Table A3 in the Supporting Information. We also present correlations between the variables in Table A4 in the Supporting Information, where we find that residing on the border with Ukraine is negatively correlated with working choice but is positively correlated with need to avoid unsafe areas.

## 4 Results

### 4.1 Uncertainty and Labor Choice

This section examines how conflict uncertainty leads individuals to adjust their labor supply (i.e., **Hypothesis I**). To that end, we estimate Equation (7) using Probit regressions, with the dependent variable being a binary indicator of whether the survey respondent has worked for pay in the last seven days. Table 2 reports the results of such a regression, with the marginal effects estimated at the mean under robust standard errors.

**TABLE 2:** Baseline results: *Conflict uncertainty* and *individual working choice*.

	Working CAhoice		
	(1)	(2)	(3)
Border with Ukraine × Post 2014	−0.035[Table-fn risa70308-tbl2-note-0001]	−0.040[Table-fn risa70308-tbl2-note-0001]	−0.039[Table-fn risa70308-tbl2-note-0001]
	(0.015)	(0.016)	(0.017)
Border with Ukraine	−0.009	−0.008	−0.009
	(0.010)	(0.010)	(0.012)
Post 2014	0.097[Table-fn risa70308-tbl2-note-0001]	0.099[Table-fn risa70308-tbl2-note-0001]	0.084[Table-fn risa70308-tbl2-note-0001]
	(0.008)	(0.009)	(0.009)
Interrupted Energy Supply	−0.035[Table-fn risa70308-tbl2-note-0001]	−0.032[Table-fn risa70308-tbl2-note-0001]	−0.030[Table-fn risa70308-tbl2-note-0001]
	(0.015)	(0.016)	(0.016)
Female Gender	−0.155[Table-fn risa70308-tbl2-note-0001]	−0.152[Table-fn risa70308-tbl2-note-0001]	−0.147[Table-fn risa70308-tbl2-note-0001]
	(0.007)	(0.007)	(0.007)
Age	−0.004[Table-fn risa70308-tbl2-note-0001]	−0.003[Table-fn risa70308-tbl2-note-0001]	−0.002[Table-fn risa70308-tbl2-note-0001]
	(0.000)	(0.000)	(0.000)
Marital Status	0.051[Table-fn risa70308-tbl2-note-0001]	0.048[Table-fn risa70308-tbl2-note-0001]	0.045[Table-fn risa70308-tbl2-note-0001]
	(0.007)	(0.008)	(0.008)
Born in the Same Country	0.104[Table-fn risa70308-tbl2-note-0001]	0.116[Table-fn risa70308-tbl2-note-0001]	0.103[Table-fn risa70308-tbl2-note-0001]
	(0.025)	(0.028)	(0.029)
Citizen of the Country	−0.053	−0.051	−0.057
	(0.046)	(0.049)	(0.049)
Unemployed 3 Months Plus	−0.027[Table-fn risa70308-tbl2-note-0001]	−0.030[Table-fn risa70308-tbl2-note-0001]	−0.028[Table-fn risa70308-tbl2-note-0001]
	(0.007)	(0.008)	(0.008)
Time Spent on Media	−0.030[Table-fn risa70308-tbl2-note-0001]	−0.031[Table-fn risa70308-tbl2-note-0001]	−0.020[Table-fn risa70308-tbl2-note-0001]
	(0.008)	(0.009)	(0.010)
Children at Home	0.047[Table-fn risa70308-tbl2-note-0001]	0.047[Table-fn risa70308-tbl2-note-0001]	0.018[Table-fn risa70308-tbl2-note-0001]
	(0.006)	(0.007)	(0.007)
Knowledge/skills Training	0.159[Table-fn risa70308-tbl2-note-0001]	0.162[Table-fn risa70308-tbl2-note-0001]	0.152[Table-fn risa70308-tbl2-note-0001]
	(0.008)	(0.009)	(0.009)
Permanently Sick	−0.515[Table-fn risa70308-tbl2-note-0001]	−0.543[Table-fn risa70308-tbl2-note-0001]	−0.533[Table-fn risa70308-tbl2-note-0001]
	(0.031)	(0.033)	(0.038)
State Of Economy		0.004[Table-fn risa70308-tbl2-note-0001]	0.006[Table-fn risa70308-tbl2-note-0001]
		(0.002)	(0.002)
State of Health Services		−0.006[Table-fn risa70308-tbl2-note-0001]	−0.004[Table-fn risa70308-tbl2-note-0001]
		(0.002)	(0.002)
State of Education		−0.002	−0.002
		(0.002)	(0.002)
Trust in Political Parties			−0.004
			(0.003)
Trust in Country's Parliament			−0.004[Table-fn risa70308-tbl2-note-0001]
			(0.002)
Trust in Politicians			0.004
			(0.003)
Trust in the Police			0.000
			(0.002)
Income sources FE	Yes	Yes	Yes
Occupation FE	Yes	Yes	Yes
Highest education FE	Yes	Yes	Yes
Country FE	Yes	Yes	Yes
Pseudo R‐squared	0.491	0.480	0.494
Observations	49,910	44,404	39,820

*Note*: This table presents the baseline results of Probit regressions concerning the impact of increased conflict uncertainty (i.e., due to the Russian Annexation of Crimea 2014) on Individual Working Choice. The dependent variable is Working Choice. This binary variable indicates whether an individual has worked in the last 7 days (i.e., as an employee, as a self‐employed individual, or for a family business). Border with Ukraine is a dummy variable that indicates whether an individual resides in a locality with a border with Ukraine. Post 2014 is an indicator function that takes a value of one if the observation is in or after 2014 and zero otherwise. Our interest is in the coefficients of Border with Ukraine × Post 2014. Our control variables include individual demographics, the state of the economy, the state of health services, the state of education, and trust. More details of control variables are presented in Table A1 in the supporting Information. We include income sources, occupation, highest education level, and country fixed effects. Robust standard errors are shown in parentheses. Significance levels are denoted as follows: * for *p* < 0.10, 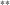 for *p* < 0.05, and *** for *p* < 0.01.

Table 2 indicates that following the Russia–Ukraine conflict in 2014, individuals who live near the border with Ukraine are 3.5–4.0 percentage points less likely to work for paid jobs than individuals who do not live near such a border. Such an interpretation is based on the marginal effects reported based on the coefficient $\beta_{1}$ on the interaction term $\text{Border with}\;{\text{Ukraine}}_{i}\times \text{Post}\;{\text{2014}}_{t}$. Given that the coefficient on $\text{Border with}\;{\text{Ukraine}}_{i}$ is statistically insignificant across all specifications, individuals residing near the border do not appear systematically different from those farther away prior to treatment. Moreover, our later analysis in Table 8 confirms the absence of differential pre‐trends, as the coefficients on the pre‐2014 interaction terms are largely statistically indistinguishable from zero, and the divergence between border and non‐border regions emerges robustly only after 2014. As a result, our results in Table 2 indicate that conflict uncertainty (i.e., living near the border with Ukraine after 2014) induces respondents to decrease their supply of labor.

**TABLE 3:** *Conflict uncertainty* and *individual perspective of safety*

	Need to Avoid Unsafe Areas		
	(1)	(2)	(3)
Border with Ukraine × Post 2014	0.023[Table-fn risa70308-tbl3-note-0001]	0.025[Table-fn risa70308-tbl3-note-0001]	0.025[Table-fn risa70308-tbl3-note-0001]
	(0.007)	(0.007)	(0.007)
Border with Ukraine	0.009[Table-fn risa70308-tbl3-note-0001]	0.007	0.005
	(0.004)	(0.004)	(0.005)
Post 2014	−0.044[Table-fn risa70308-tbl3-note-0001]	−0.039[Table-fn risa70308-tbl3-note-0001]	−0.040[Table-fn risa70308-tbl3-note-0001]
	(0.003)	(0.003)	(0.004)
Demographic controls	Yes	Yes	Yes
State controls	No	Yes	Yes
Trust controls	No	No	Yes
Income sources FE	Yes	Yes	Yes
Occupation FE	Yes	Yes	Yes
Highest education FE	Yes	Yes	Yes
Country FE	Yes	Yes	Yes
Pseudo R‐squared	0.022	0.024	0.026
Observations	46,889	41,680	37,204

*Note*: This table presents the results of Probit regressions concerning the impact of increased conflict uncertainty on individual perception of safety. The dependent variable is NAeed to avoid Unsafe Areas, a binary variable indicating whether subjective risk aversion has increased for an individual in avoiding unsafe areas to live in. Border with Ukraine is a dummy variable that indicates whether an individual resides in a locality with a border with Ukraine. Post 2014 is an indicator function that takes a value of one if the observation is in or after 2014 and zero otherwise. Our interest is in the coefficients of border with Ukraine × Post 2014. Our control variables include individual demographics, the state of the economy, the state of health services, the state of education, and trust. More details of control variables are presented in Table A1 in the Supporting Information. We include income sources, occupation, highest education level, and country fixed effects. Robust standard errors are shown in parentheses. Significance levels are denoted as follows: * for *p* < 0.10, 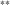 for *p* < 0.05, and *** for *p* < 0.01.

**TABLE 4:** *conflict uncertainty* and *individual working choice* under perspective of safety.

	Working Choice (need to avoid unsafe areas = 1)			Working Choice (need to avoid unsafe areas = 0)		
	(1)	(2)	(3)	(4)	(5)	(6)
Border with Ukraine × Post 2014	−0.045[Table-fn risa70308-tbl4-note-0001]	−0.052[Table-fn risa70308-tbl4-note-0001]	−0.051[Table-fn risa70308-tbl4-note-0001]	0.016	0.021	0.037
	(0.017)	(0.018)	(0.019)	(0.059)	(0.061)	(0.064)
Border with Ukraine	−0.010	−0.007	−0.008	−0.011	−0.035	−0.044
	(0.010)	(0.011)	(0.012)	(0.039)	(0.041)	(0.045)
Post 2014	0.089[Table-fn risa70308-tbl4-note-0001]	0.091[Table-fn risa70308-tbl4-note-0001]	0.077[Table-fn risa70308-tbl4-note-0001]	0.065[Table-fn risa70308-tbl4-note-0001]	0.075[Table-fn risa70308-tbl4-note-0001]	0.049
	(0.009)	(0.009)	(0.010)	(0.029)	(0.030)	(0.031)
Demographic Controls	Yes	Yes	Yes	Yes	Yes	Yes
State Controls	No	Yes	Yes	No	Yes	Yes
Trust Controls	No	No	Yes	No	No	Yes
Income Sources FE	Yes	Yes	Yes	Yes	Yes	Yes
Occupation FE	Yes	Yes	Yes	Yes	Yes	Yes
Highest Education FE	Yes	Yes	Yes	Yes	Yes	Yes
Country FE	Yes	Yes	Yes	Yes	Yes	Yes
Pseudo R‐squared	0.497	0.486	0.501	0.460	0.449	0.466
Observations	43,141	38,325	34,160	3,748	3,355	3,044

*Note*: This table presents the results of Probit regressions concerning the impact of increased conflict uncertainty on individual working choice, across different degrees of risk aversion. The dependent variable is Working Choice. This binary variable indicates whether an individual has been working in the past 7 days (i.e., employed, self‐employed, or working in a family business). Border with Ukraine is a dummy variable that indicates whether an individual resides in a locality with a border with Ukraine. Post 2014 is an indicator function that takes a value of one if the observation is in or after 2014 and zero otherwise. Our interest is in the coefficients of Border with Ukraine × Post 2014. Columns (1)–(3) use a subsample where the value of Need to Avoid Unsafe Areas is one, meaning the individuals are risk‐averse (to avoid unsafe areas to live). In contrast, Columns (4)–(6) use a subsample where the value of Need to Avoid Unsafe Areas is zero, meaning the individuals are not risk‐averse. Our control variables include individual demographics, economic conditions, health service conditions, educational conditions, and trust. More details of control variables are presented in Table A1 in the Supporting Information. We include income sources, occupation, highest education level, and country fixed effects. Robust standard errors are shown in parentheses. Significance levels are denoted as follows: * for *p* < 0.10, 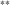 for *p* < 0.05, and *** for *p* < 0.01.

**TABLE 5:** Robustness tests: Alternative working variable

	Working Hours (log)		
	(1)	(2)	(3)
Border with Ukraine × Post 2014	−0.021[Table-fn risa70308-tbl5-note-0001]	−0.027[Table-fn risa70308-tbl5-note-0001]	−0.026[Table-fn risa70308-tbl5-note-0001]
	(0.010)	(0.011)	(0.011)
Border with Ukraine	−0.011[Table-fn risa70308-tbl5-note-0001]	−0.008	−0.008
	(0.005)	(0.005)	(0.006)
Post 2014	0.095[Table-fn risa70308-tbl5-note-0001]	0.098[Table-fn risa70308-tbl5-note-0001]	0.097[Table-fn risa70308-tbl5-note-0001]
	(0.005)	(0.006)	(0.006)
Demographic Controls	Yes	Yes	Yes
State Controls	No	Yes	Yes
Trust Controls	No	No	Yes
Income sources FE	Yes	Yes	Yes
Occupation FE	Yes	Yes	Yes
Highest Education FE	Yes	Yes	Yes
Country FE	Yes	Yes	Yes
Pseudo R‐squared	0.055	0.058	0.056
Observations	41,312	37,268	33,734

*Note*: This table presents the results of Tobit regressions on the impact of conflict uncertainty on individual working hours. The dependent variable is Working Hours (Log), which is the natural logarithm of one plus the total contracted working hours per week in the main job. From Table 1, the maximum recorded value for Working Hours (raw) is 555, which is well larger than the plausible number of hours per week. To account for outliers (e.g., 555 h), we estimate a Tobit model with an upper bound of 5.13, corresponding to the logarithm of 168 h, the maximum number of hours available per week. Border with Ukraine is a dummy variable that indicates whether an individual resides in a locality with a border with Ukraine. Post 2014 is an indicator function that takes a value of one if the observation is in or after 2014 and zero otherwise. Our interest is in the coefficients of Border with Ukraine × Post 2014. Our control variables include individual demographics, the state of the economy, the state of health services, the state of education, and trust. More details of control variables are presented in Table A1 in the Supporting Information. We include income sources, occupation, highest education level, and country fixed effects. Robust standard errors are shown in parentheses. Significance levels are denoted as follows: * for *p* < 0.10, 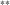 for *p* < 0.05, and *** for *p* < 0.01.

**TABLE 6:** Robustness: Alternative clustering for standard errors

	Working Choice			
	(1)	(2)	(3)	(4)
Border with Ukraine × Post 2014	−0.039[Table-fn risa70308-tbl6-note-0001]	−0.039[Table-fn risa70308-tbl6-note-0001]	−0.039[Table-fn risa70308-tbl6-note-0001]	−0.039[Table-fn risa70308-tbl6-note-0001]
	(0.017)	(0.017)	(0.017)	(0.017)
Border with Ukraine	−0.009	−0.009	−0.009	−0.009
	(0.012)	(0.012)	(0.012)	(0.012)
Post 2014	0.084[Table-fn risa70308-tbl6-note-0001]	0.084[Table-fn risa70308-tbl6-note-0001]	0.084[Table-fn risa70308-tbl6-note-0001]	0.084[Table-fn risa70308-tbl6-note-0001]
	(0.009)	(0.009)	(0.009)	(0.009)
Demographic Controls	Yes	Yes	Yes	Yes
State Controls	Yes	Yes	Yes	Yes
Trust Controls	Yes	Yes	Yes	Yes
Income Sources FE	Yes	Yes	Yes	Yes
Occupation FE	Yes	Yes	Yes	Yes
Highest Education FE	Yes	Yes	Yes	Yes
Country FE	Yes	Yes	Yes	Yes
Pseudo R‐squared	0.494	0.494	0.494	0.494
Observations	39,820	39,820	39,820	39,820
Clusters	Robust	ID × Country	ID × Region	ID × ESS Round

*Note*: This table presents the results of Probit regressions concerning how increased conflict uncertainty impacts individual working choice. Unlike the baseline regressions, we use different clusters for standard errors. Column (1) reproduces the baseline Probit regression. In Column (2), standard errors are clustered at the participant and country levels, while in Column (3), they are clustered at the participant and region levels. Column (4) reports standard errors clustered at the participant and ESS round levels. The standard errors are shown in parentheses. The dependent variable is working choice, a binary variable that captures whether an individual has been working for the last seven days (i.e., employee, self‐employed, working for your family business). Border with Ukraine is a dummy variable that indicates whether an individual resides in a locality on the Ukrainian border. Post 2014 is an indicator function that takes a value of one if the observation is in or after 2014 and zero otherwise. Our interest is in the coefficients of border with Ukraine × Post 2014. Our control variables include individual demographics, the state of the economy, the state of health services, the state of education, and trust. More details of control variables are presented in Table A1 in the Supporting Information. We include income sources, occupation, highest education level, and country fixed effects. Significance levels are denoted as follows: * for *p* < 0.10, 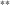 for *p* < 0.05, and *** for *p* < 0.01.

**TABLE 7:** Robustness: Additional control variables

	Working CChoice		
	(1)	(2)	(3)
Border with Ukraine × Post 2014	−0.036[Table-fn risa70308-tbl7-note-0001]	−0.036[Table-fn risa70308-tbl7-note-0001]	−0.035[Table-fn risa70308-tbl7-note-0001]
	(0.018)	(0.018)	(0.018)
Border with Ukraine	−0.012	−0.012	−0.012
	(0.012)	(0.012)	(0.012)
Post 2014	0.082[Table-fn risa70308-tbl7-note-0001]	0.086[Table-fn risa70308-tbl7-note-0001]	0.088[Table-fn risa70308-tbl7-note-0001]
	(0.009)	(0.009)	(0.009)
Immigrants Making the Country Better	−0.001	−0.001	−0.001
	(0.002)	(0.002)	(0.002)
Breathing Problem		−0.080[Table-fn risa70308-tbl7-note-0001]	−0.080[Table-fn risa70308-tbl7-note-0001]
		(0.039)	(0.039)
Noise Problem		−0.041[Table-fn risa70308-tbl7-note-0001]	−0.041[Table-fn risa70308-tbl7-note-0001]
		(0.024)	(0.024)
Local Engagement			0.099[Table-fn risa70308-tbl7-note-0001]
			(0.027)
Demographic Controls	Yes	Yes	Yes
State Controls	Yes	Yes	Yes
Trust Controls	Yes	Yes	Yes
Income Sources FE	Yes	Yes	Yes
Occupation FE	Yes	Yes	Yes
Highest Education FE	Yes	Yes	Yes
Country FE	Yes	Yes	Yes
Pseudo R‐squared	0.496	0.496	0.496
Observations	37,546	37,546	37,546

*Note*: This table presents results from Probit regressions examining how increased conflict uncertainty affects individual work choice. In this table, we include additional control variables. In addition to the baseline controls, Column (1) includes perceptions of immigrants. Column (2) includes measures of breathing problems and noise problems to capture air and noise quality. Column (3) includes local engagement, which measures whether survey participants participate in local activities such as religious events, social clubs, or volunteer activities. The standard errors are shown in parentheses. The dependent variable is working choice, a binary variable that captures whether an individual has been working for the last seven days (i.e., employee, self‐employed, working for your family business). Border with Ukraine is a dummy variable that indicates whether an individual resides in a locality on the Ukrainian border. Post 2014 is an indicator function that takes a value of one if the observation is in or after 2014 and zero otherwise. Our interest is in the coefficients of border with Ukraine × Post 2014. Our control variables include individual demographics, the state of the economy, the state of health services, the state of education, and trust. More details of control variables are presented in Table A1 in the Supporting Information. We include income sources, occupation, highest education level, and country fixed effects. Significance levels are denoted as follows: * for *p* < 0.10, 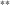 for *p* < 0.05, and *** for *p* < 0.01.

**TABLE 8:** Robustness: Regression individual year dummies

	Working Choice					
	(1)	(2)	(3)	(4)	(5)	(6)
Border with Ukraine × Year 2004	0.018	0.051	0.062	0.011	0.043	0.054
	(0.035)	(0.046)	(0.049)	(0.038)	(0.049)	(0.044)
Border with Ukraine × Year 2005				0.037	0.054	0.056
				(0.048)	(0.051)	(0.046)
Border with Ukraine × Year 2006	0.057[Table-fn risa70308-tbl8-note-0001]	0.051	0.052	0.050	0.044	0.045
	(0.031)	(0.033)	(0.036)	(0.035)	(0.037)	(0.029)
Border with Ukraine × Year 2007				−0.078	−0.070	−0.063
				(0.051)	(0.054)	(0.049)
Border with Ukraine × Year 2008	−0.032	−0.020	−0.019	−0.038	−0.026	−0.026
	(0.035)	(0.036)	(0.039)	(0.038)	(0.040)	(0.032)
Border with Ukraine × Year 2009				−0.041	−0.058	−0.052
				(0.039)	(0.041)	(0.033)
Border with Ukraine × Year 2010	−0.050[Table-fn risa70308-tbl8-note-0001]	−0.049	−0.046	−0.056[Table-fn risa70308-tbl8-note-0001]	−0.055	−0.052[Table-fn risa70308-tbl8-note-0001]
	(0.029)	(0.030)	(0.034)	(0.033)	(0.035)	(0.026)
Border with Ukraine × Year 2011				−0.023	−0.009	−0.001
				(0.072)	(0.076)	(0.073)
Border with Ukraine × Year 2013				0.102	0.101	0.117[Table-fn risa70308-tbl8-note-0001]
				(0.068)	(0.071)	(0.067)
Border with Ukraine × Post 2014	−0.040[Table-fn risa70308-tbl8-note-0001]	−0.045[Table-fn risa70308-tbl8-note-0001]	−0.041[Table-fn risa70308-tbl8-note-0001]	−0.046[Table-fn risa70308-tbl8-note-0001]	−0.051[Table-fn risa70308-tbl8-note-0001]	−0.048[Table-fn risa70308-tbl8-note-0001]
	(0.019)	(0.020)	(0.025)	(0.025)	(0.026)	(0.013)
Demographic Controls	Yes	Yes	Yes	Yes	Yes	Yes
State Controls	No	Yes	Yes	No	Yes	Yes
Trust Controls	No	No	Yes	No	No	Yes
Income sources FE	Yes	Yes	Yes	Yes	Yes	Yes
Occupation FE	Yes	Yes	Yes	Yes	Yes	Yes
Highest Education FE	Yes	Yes	Yes	Yes	Yes	Yes
Country FE	Yes	Yes	Yes	Yes	Yes	Yes
Pseudo R‐squared	0.491	0.481	0.495	0.491	0.481	0.495
Observations	49,910	44,404	39,820	49,910	44,404	39,820

*Note*: This table presents the results of Probit regressions concerning the impact of increased conflict uncertainty (i.e., due to the Russian Annexation of Crimea 2014) on individual working choice. The dependent variable is working choice. This binary variable indicates whether an individual has worked in the last 7 days (i.e., as an employee, as a self‐employed person, or for a family business). Border with Ukraine is a dummy variable that indicates whether an individual resides in a locality with a border with Ukraine. We interact Border with Ukraine with a dummy variable Yeart, where *t* is one of the years from 2004 to 2013, except 2012. Columns (1)–(3) use the ESS wave years, while Columns (4)–(6) use the interview years. Yeart equals one if the observed year is exactly year t, and zero otherwise. Post 2014 is an indicator function that takes a value of one if the observation is in or after 2014 and zero otherwise. We also include non‐interaction variables, including Border with Ukraine, Post 2014, and all dummies of Yeart. The year 2012 is omitted as the baseline; Year 2002 is dropped due to limited country coverage in that wave. For brevity, we do not report their coefficients. We use robust standard errors and show them in parentheses. Significance levels are denoted as follows: * for *p* < 0.10, 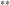 for *p* < 0.05, and *** for *p* < 0.01.

We note that the estimated coefficient for $\beta_{1}$ is statistically significant even after controlling for whether the survey respondent is permanently sick/disabled, unemployed, or caring for dependents. Such consistent significance indicates that our results reported in Table 2 are not driven by the possibility that the individual is sick and therefore is unable to work, the possibility that the individual is simply unemployed, or the possibility that they need to cut their working hours due to the need to take care of their dependents. In addition, we find that the results are robust to controlling for overall economic conditions, individuals' demographic characteristics, income, education level, and their perception of their country of residence.

Given that conflicts often result in fatalities, environmental damage, and a decline in overall safety, heightened uncertainty surrounding conflicts suggests a potential rise in HS&E risks. Consequently, the results reported in Table 2 can also be interpreted as the negative impacts of increased HS&E risks stemming from conflict uncertainty on labor‐supply choices.

### 4.2 Increased Risk Aversion, Uncertainty, and Labor Supply

Having documented that uncertainty arising from conflicts reduces individuals' labor supply (i.e., **Hypothesis I**), we next consider how such uncertainty can affect these individuals' subjective risk aversion and, in turn, their labor supply choice (i.e., **Hypothesis II**). To that end, we estimate Equation (8) using a series of Probit regressions, with the dependent variable a binary indicator of whether the survey respondent reports an increase in the need to avoid unsafe areas. Table 3 reports the results of such a regression, with the marginal effects estimated at the mean under robust standard errors.

To economize on space, Table 3 uses the exact set of controls as in Table 2, but only indicates these controls underneath the estimated coefficients. Specifically, we divide our set of controls in Table 2 into three groups: demographic, state, and trust controls.^21^ We then indicate under Table 3 whether we control for these three groups in each column. Going forward, we will adopt this convention throughout the remaining tables.

Our coefficient of interest in Table 3 is $\beta_{1}$, which captures the effects of the interaction term $\text{Border with}\;{\text{Ukraine}}_{i}\times \text{Post}\;{\text{2014}}_{t}$. The statistically significant and positive estimates of $\beta_{1}$ in Table 3 indicate that individuals who live near the border with Ukraine are subject to an increase of roughly 2.5% in the need to avoid unsafe areas post‐2014 relative to their counterparts who are outside of the border regions. Given that the need to avoid unsafe areas reflects individual risk aversion, our results indicate that living near the border induces survey respondents to increase their risk aversion post‐2014 relative to those in other regions. Our finding of increased risk aversion following conflict risk aligns with prior empirical evidence (e.g., Callen et al. 2014).

We find it reassuring that individuals living near the border are ex ante similar to individuals located outside of the border regions, as evidenced by the insignificance of the coefficient on border with Ukraine (i.e., $\beta_{2}$). In other words, our results on the positive estimates of $\beta_{1}$ were not driven by the possibility that these two groups are ex ante different from each other. In addition, our estimates of $\beta_{1}$ are consistent regardless of whether the controls are included. Such continued significance indicates that our result, vis‐à‐vis the increase in risk aversion among individuals exposed to conflict uncertainty relative to non‐exposed individuals, is not driven by their demographic differences, the state of the economy in which they reside, or their trust in the institution.

We also note that our dependent variable of interest in Table 3, need to avoid unsafe areas, is unlikely to reflect broader safety preferences or other local factors, such as crime or policing. First, our regression specification absorbs any time‐invariant differences in crime rates, policing quality, or baseline safety preferences across border and non‐border regions. The coefficient of interest thus captures only the differential change in reported safety orientation for border regions post 2014. For crime or policing to confound our estimates, such factors would need to have shifted differentially between border and non‐border regions at the onset of the 2014 conflict. We are not aware of evidence suggesting that crime rates or policing practices experienced such a border‐specific change in 2014. Second, our specification controls for trust in the police, a direct proxy for individuals' perceptions of policing quality. The inclusion of this control partially accounts for the possibility that changes in safety perceptions reflect shifts in confidence about law enforcement rather than conflict‐related risk aversion.

Having confirmed that conflict uncertainty affects an individual's subjective risk aversion, we next examine whether the labor supply response to conflict uncertainty differs between individuals who report an increase in risk aversion and those who do not. To that end, Table 4 presents the estimates of Equation (7) for these two groups.

Specifically, Columns (1)–(3) report the estimates of Equation (7) for individuals reporting an increase in risk aversion, whereas Columns (4)–(6) report the estimates for individuals reporting no such increase. We find that individuals reporting an increase in risk aversion reduce their labor supply in response to conflict uncertainty (i.e., the estimate for $\beta_{1}$ is negative and statistically significant regardless of which controls are included). In stark contrast, we document no statistically significant effects of conflict uncertainty on working choice for individuals who report no change in risk aversion across all specifications.^22^

Such a dichotomy is consistent with the increase in perceived risk aversion as a potential mechanism that explains how individuals reduce their labor supply following an increase in conflict uncertainty arising from Russia's 2014 invasion of Ukraine. In other words, these results are consistent with **Hypothesis II** in the sense that the resulting increase in risk aversion drives the reported fall in labor supply in response to conflict uncertainty. In the context of the related literature, this result provides evidence supporting seminal works by Kahneman (Kahneman and Tversky 1974; 1979; Tversky and Kahneman 1981; 1991), which show that changes in risk perception lead individuals to alter the types of activities they aspire to engage in.

Table 4 also provides direct evidence that conflict uncertainty, rather than other confounding factors, drives our results. The negative labor supply response is concentrated entirely among individuals reporting increased perceived risk aversion. If other factors, such as cultural ties or business connections, were responsible for such results, we would expect similar effects across both groups. The absence of a statistically significant effect among individuals with unchanged risk perceptions suggests that the activated loss‐aversion channel, rather than pre‐existing regional characteristics, explains the documented labor supply response.^23^

Our results on the change in perceived and revealed risk aversion (i.e., labor choice) following increased conflict uncertainty are robust after controlling for various demographic characteristics. These characteristics, for example, gender (Jianakoplos and Bernasek 1998; Schubert et al. 1999; Borghans et al. 2009), age (Bellante and Green 2004; Hanoch et al. 2018), employment status (Chetty and Szeidl 2007; Backus and Esteller‐Moré 2017), the time the individual typically spends on media (Allcott et al. 2020), and marital status (Roussanov and Savor 2014), among others, have been shown to impact individual intrinsic risk aversion. In other words, our results documented in this section are not simply artifacts of individual intrinsic risk aversion.

### 4.3 Robustness

In this section, we conduct several exercises to check the robustness of our results. First, instead of using a binary indicator of whether an individual works in paid employment, we use an alternative measure of the individual's total weekly working hours. Second, the region of residence is randomized. Next, we also randomize the timing of the conflict. Randomization allows us to assess whether incorrect location identification and conflict timing in our empirical strategy drive our results. Finally, we employ different clusters for standard errors in our model specifications. Overall, our results are consistent across all of these specifications.^24^

#### 4.3.1 Alternative Measures of Labor

In Equation (7), we model labor choice using a binary variable that equals 1 if the individual worked in paid employment in the last week and 0 otherwise. It may be argued that the individual choice to work or not may struggle to fully capture the veracity of the labor supply. Therefore, in this section, we employ a continuous variable that captures total contracted hours per week in the main job as an alternative measure of labor supply.^25^ We employ a Tobit specification as the working hours are constrained by the number of available hours per week.^26^

(9) $$\begin{array}{ccc} \text{Working Hours}\;{\text{(log)}}_{i,t} & = & \alpha +\beta_{1}\text{Border with}\;{\text{Ukraine}}_{i}\times \text{Post}\;{\text{2014}}_{t}\\ & & +\;\beta_{2}\text{Border with}\;{\text{Ukraine}}_{i}+\beta_{3}\text{Post}\;{\text{2014}}_{t}\\ & & +\;\zeta X_{i,t}+\lambda_{c}+\varphi_{s}+\delta_{o}+\gamma_{e}+\varepsilon_{i,t}, \end{array}$$

where $\text{Working Hours}\;{\text{(log)}}_{i,t}$ is the natural logarithm of one plus the total contracted hours per week in the main job. $\text{Border with}\;{\text{Ukraine}}_{i}$ is a binary variable that indicates whether individual i resides in a locality that shares border with Ukraine, and $\text{post}\;{\text{2014}}_{t}$ is an indicator function that takes a value of one if the observation is in or after 2014 and zero otherwise. $X_{i,t}$ denotes the same set of control variables defined in Equation (7). $\lambda_{c}$, $\varphi_{s}$, $\delta_{o}$, and $\gamma_{e}$ are the country, income source, occupation, and highest education level fixed effects, respectively. The specification employs robust standard errors. $\beta_{1}$ is the coefficient of interest, capturing how working hours are affected among individuals residing on the border with Ukraine after the 2014 Russia–Ukraine conflict.

We present our results in Table 5, which show that $\beta_{1}$ is negative and significant across all columns (including control variables for individual demographic characteristics, the state of the economy, health services, education, and trust). In terms of economic magnitude, individuals residing in regions bordering Ukraine after 2014 reduce their working hours by 2.1%–2.7% relative to their counterparts in non‐border regions. Even though the significance of the coefficients of $\text{Border with}\;{\text{Ukraine}}_{i}$ reduces with the inclusion of additional control variables, the coefficients are qualitatively consistent with our benchmark results in Table 2. We also conduct Equation (9) with different clusters for standard errors and present the results in Table A7 in the Supporting Information, where we find that the estimated effects according to the interaction term $\text{Border with}\;{\text{Ukraine}}_{i}\times \text{Post}\;{\text{2014}}_{t}$ are robust to these clustering strategies.

The results in this section are consistent with those from Equation (7), which estimated the likelihood of working in paid jobs. Our results with the alternative measure of labor supply reconfirm the impact of risk on labor decisions, consistent with the theory of Kahneman and Tversky (1974;1979) and Tversky and Kahneman (1981;1991). In our context, given the uncertainties inherent in the geopolitical conflict between Russia and Ukraine, particularly since 2014, which are related to HS&E risks (Pereira et al. 2022; Lewtak et al. 2022), the documented effects can be interpreted as attributable to a potential increase in HS&E risks.

#### 4.3.2 Placebo Tests: Randomizing Locations

**FIGURE 4:**
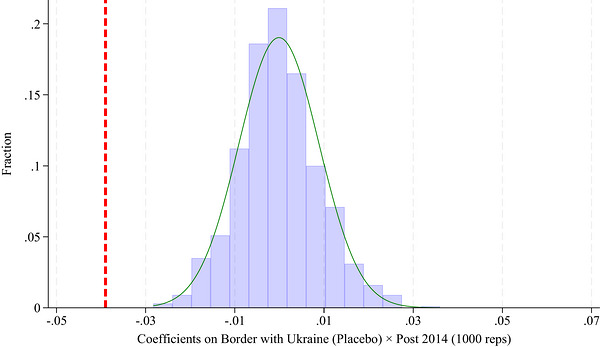
Estimates using randomized border with Ukraine. *Note*: This figure displays a placebo test using a placebo measurement *Border with Ukraine (Placebo)* instead of the actual *Border with Ukraine*. We repeat the randomization 1000 times and report the distribution of the estimated coefficients on Border with Ukraine (Placebo) × Post 2014. The dependent variable is Working Choice. The true estimate, using the actual *Border with Ukraine* × Post 2014 from our baseline result, is overlaid as a red vertical line in the figure.

This section examines whether our main results are driven by misidentifying the locations bordering Ukraine. Therefore, we re‐estimate Equation (7), but instead of using the original variable—border with ${\text{Ukraine}}_{i}$, we randomly draw the individuals' regions from a uniform distribution and create a new variable—border with $\text{Ukraine}\;{\text{(placebo)}}_{i}$. We conduct the randomization 1000 times for $\text{Border with Ukraine}\;{\text{(placebo)}}_{i}$ and present our results in Figure 4.

Figure 4 shows the distribution of the placebo estimates for the interaction term across 1000 replications. Figure 4 also overlays the true estimate denoted by the vertical line in Figure 4. The fact that the true estimate lies beyond the tail of the placebo distribution suggests that our baseline findings are not driven by random variation in our proximity measure.

As our baseline regressions focus on countries that are near the Ukrainian border, individuals in countries such as Estonia, Finland, Latvia, and Lithuania, which directly border Russia, may also be affected. To address this concern, we construct an additional binary variable, border with Russia, which equals 1 if a region borders Russia and 0 otherwise.^27^ We interact this indicator with the Post‐2014 variable and report the results in Table A6 in the Supporting Information. Overall, we find no evidence that the 2014 Russia–Ukraine conflict affected individuals' labor choices in regions bordering Russia.

#### 4.3.3 Placebo Tests: Randomizing Conflict Timing

Next, we examine whether the timing of the conflict's choice can account for our baseline results. Instead of using the actual year of the Russia–Ukraine conflict in 2014 in our Equation (7), in this section, we randomly assign the conflict year from 2002 to 2023 to create $\text{Post}\;{\text{(placebo)}}_{t}$, repeating this process 1000 times.

**FIGURE 5:**
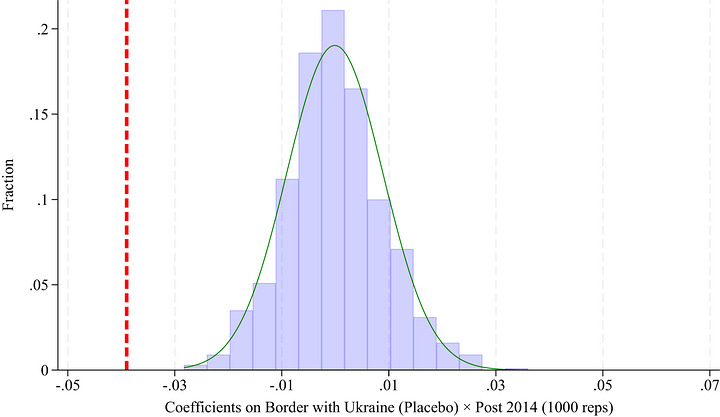
Estimates using randomized timing. *Note*: This figure displays a placebo test using a placebo measurement *Post (Placebo)* instead of the actual *Post 2014*. We repeat the randomization 1000 replications and report the distribution of the estimated coefficients on Border with Ukraine × Post (Placebo). The dependent variable is Working Choice. The true estimate, using the actual *Border with Ukraine* × Post 2014 from our baseline result, is overlaid as a red vertical line in the figure.

In Figure 5, the distribution of estimates for the interaction term between border with ${\text{Ukraine}}_{i}$ and $\text{Post}\;{\text{(placebo)}}_{t}$ is shown across 1000 replications. The vertical line represents the true coefficient of the interaction term between border with ${\text{Ukraine}}_{i}$ and $\text{Post}\;{\text{2014}}_{t}$, which is distant from the distribution of the placebo interaction term's coefficients. These results suggest that the timing of the 2014 conflict is not random and that our baseline results are not driven by misidentification of the conflict year.

We shift the timing by one, two, and three years before 2014 to test whether the choice of the 2014 conflict is random. The results are shown in Table A8 in the Supporting Information. Additionally, instead of randomizing the conflict timing using years between 2002 and 2014, as shown in Figure 5, we randomize the conflict timing using only the years before 2014 (i.e., 2002–2013), with the results presented in Figure A1 in the Supporting Information. Overall, our baseline results remain consistent even after conducting different exercises to test the timing choice of the conflict.

#### 4.3.4 Different Clusters for Standard Errors

In this section, we re‐estimate Equation (7) using alternative clustering levels. The results are reported in Table 6. Column (1) reproduces the baseline Probit regression. In Column (2), standard errors are clustered at the participant and country levels, while in Column (3), they are clustered at the participant and region levels. Finally, Column (4) reports standard errors clustered at the participant and ESS round levels. Despite these differences in clustering levels, the results remain qualitatively similar to the baseline specification.

#### 4.3.5 Additional Controls

It is possible that the observed risk may arise from the Russia–Ukraine conflict itself or instead reflect other pre‐existing factors. In this section, we therefore include additional controls for respondents' perceptions of immigrants, air quality, and noise quality. These controls help account for local attitudes and environmental conditions that may be correlated with both perceived risk and our outcomes of interest.

We also seek to capture whether respondents in the ESS are likely to have relocated. Unfortunately, the ESS does not directly ask whether respondents moved to another area. In addition, the survey asks how long respondents have lived in their area, only in the 2002–2003 wave. As a proxy for residential stability in other waves, we use indicators of local integration, such as participation in local churches, involvement in local social activities, and attendance at local events. These measures capture respondents' physical and social engagement with their area, which has been shown to be positively correlated with residence tenure (see, e.g., Keene et al. 2013; Leviten‐Reid and Matthew 2017; Gepty et al. 2022).

Including these additional variables in our regression specifications does not materially affect our baseline results. We report the corresponding estimates in Table 7.^28^

#### 4.3.6 Parallel Trends: A Sensitivity Analysis

We estimate a regression specification in which we interact $\text{border with}\;{\text{Ukraine}}_{i}$ with ESS wave and year indicators

(10) $$\begin{array}{ccc} \text{Working}\;{\text{Choice}}_{i,t} & = & \alpha +\sum_{t<2014}\beta_{t}\;\text{Border with}\;{\text{Ukraine}}_{i}\times I_{t}\\ & & +\;\beta_{\text{post}}\text{Border with}\;{\text{Ukraine}}_{i}\times \text{Post}\;{\text{2014}}_{t}\\ & & +\;\beta_{\text{2014}}\text{Post}\;{\text{2014}}_{t}+\beta_{U}\text{Border with}\;{\text{Ukraine}}_{i}\\ & & +\;\sum_{t<2014}\gamma_{t}I_{t}+\zeta X_{i,t}+\lambda_{c}+\varphi_{s}+\delta_{o}+\gamma_{e}+\varepsilon_{i,t}, \end{array}$$

where $I_{t}$ denotes indicator functions for each ESS wave (or interview year). This hybrid specification allows us to examine pre‐trends on a wave‐by‐wave and year‐by‐year basis while preserving statistical power for the treatment effect by pooling the post‐2014 observations. Such an approach to combining disaggregated leads with a binned post‐treatment effect is well established in the event study literature (see, e.g., Schmidheiny and Siegloch 2023).

We report the results in Table 8 without the noninteraction terms (i.e., $\beta_{\text{2014}}\text{Post}\;{\text{2014}}_{t}+\beta_{U}\text{Border with}\;{\text{Ukraine}}_{i}+\sum_{t<2014}\gamma_{t}I_{t}$) for presentational convenience. In Table 8, Specifications (1)–(3) use ESS wave indicators, which are available for selected years only, whereas Specifications (4)–(6) use the interview year indicator. We find that the pre‐2014 interaction coefficients are generally small in magnitude and, overall, statistically indistinguishable from zero, providing no evidence of a persistent or monotonic differential pre‐trend between border and non‐border regions prior to the conflict.^29^ The pooled post‐treatment coefficient, $\beta_{\text{post}}$, is negative and significant, with a magnitude largely consistent with our baseline estimates of 3.5–4.0 percentage‐point reductions in the probability of survey respondents supplying labor in the presence of conflict uncertainty.^30^

## 5 Conclusion

Motivated by the seminal theory of Daniel Kahneman and Amos Tversky (Kahneman and Tversky 1974;1979; Tversky and Kahneman 1981;1991), we use the unique context of the 2014 Russia–Ukraine conflict to examine whether individuals choose to work and how they perceive the associated risks. In this setting, the geopolitical event not only causes potential damages and deaths to Ukraine and Russia but also affects residents in other countries (Liadze et al. 2023), especially neighboring nations that share borders with Ukraine. Thus, individuals residing in regions bordering Ukraine would face significant risks to their HS&E (Pereira et al. 2022; Lewtak et al. 2022) due to increased risks of nuclear power accidents, missile strikes, infectious diseases among immigrants, and air pollution. Because individuals in these neighboring countries (PHS) have not previously experienced such geopolitical risks, according to risk theory (Kahneman and Tversky 1974;1979; Tversky and Kahneman 1981;1991), those in border regions are expected to change their individual actions.

Indeed, we showed that individuals residing in border regions who face conflict risks are more likely to reduce their working hours, controlling for various economic, financial, and demographic factors. Such a reduction in labor supply can be explained by individuals becoming more risk‐averse and avoiding unsafe areas. This documented increase in perceived risk aversion is consistent with a behavioral response to the conflict, where individuals become more sensitive to potential negative outcomes. Our results are robust across different placebo tests and an alternative measure of labor supply. From a policy perspective, they highlight the need for timely support from governments and policymakers for residents in regions more directly affected by the Russia–Ukraine conflict.

## Conflicts of Interest

The author declare no conflicts of interest.

## Supporting information


**Supporting File**: supp‐0001‐SuppMat.pdf

## Endnotes

1 See, for example, Barberis (2013) for a comprehensive review of prospect theory applications in economics.

2 See, for example, DellaVigna (2009) for a comprehensive review of the related literature on how psychological factors shift preference.

3 We note that, even though Pereira et al. (2022) and Lewtak et al. (2022) focus on a later conflict, these papers provide support for the nature of modern warfare risks generally.

4 Source: https://www.ft.com/content/3df096cd‐5a0d‐4632‐be5d‐753faa573b40. Last accessed: November 10, 2025.

5 Source: https://news.un.org/en/story/2025/09/1165838. Last accessed: November 10, 2025.

6 Source: https://www.ft.com/content/0dc73556‐67f2‐4f48‐b0ad‐53f183a05df3. Last accessed: November 10, 2025.

7 Source: https://euromaidanpress.com/2025/08/21/massive‐russian‐air‐attack‐hits‐ukraine‐with‐574‐drones‐and‐40‐missiles‐including‐city‐near‐hungarian‐border/. Last accessed: November 10, 2025.

8 More broadly, this tension relates to evidence that uncertainty shocks can have meaningful real effects (Jurado et al. 2015; Baker et al. 2016; Vu and Wu 2020; Caldara and Iacoviello 2022). Our setting differs in that it focuses on a conflict‐related HS&E risk, for which the behavioral labor‐supply response may dominate the standard precautionary response.

9 Our choice of a two‐period structure provides a clean separation between the decision margin (Period 1, where the agent chooses $L_{1}$) and the realization of consequences (Period 2, where the conflict outcome materializes). The timing structure captures the forward‐looking nature of the labor supply decision under uncertainty in the sense that the agent anticipates that working today increases exposure to conflict risk tomorrow (e.g., through commuting, separation from family, or physical presence in an at‐risk location).

10 We refer readers to Bernheim and Rangel (2009) and O'Donoghue and Sprenger (2018) for a comprehensive treatment of reference‐dependent preference models and their behavioral implications.

11 We also assume that the concavity of the leisure utility and the convexity of the risk probability function $q(L_{1})$ strictly dominate the convexity of the value function in the loss domain.

12 For brevity, Equation (3) presents the gain domain case, where $C_{1}=w_{1}L_{1}\geq R_{1}$. In the loss domain ($C_{1}<R_{1}$), the prospect theory value function in (1) implies $v\left(C_{1}-R_{1}\right)=-\lambda {\left(R_{1}-w_{1}L_{1}\right)}^{\beta }$, so that $U_{1}=-\lambda {\left(R_{1}-w_{1}L_{1}\right)}^{\beta }+\frac{{\left(1-L_{1}\right)}^{1-\sigma }}{1-\sigma }$.

13 Having the conflict risk parameter impacting only the labor side is a deliberate modeling choice that reflects the specific psychological mechanism we would like to capture. The underlying idea, motivated by the literature on decision making under fear (see, e.g., Caplin and Leahy 2001), is that conflict does not alter the agent's valuation of consumption or leisure per se. In other words, θ does not change how the agent enjoys a meal or values an hour of rest. Rather, it changes how the agent perceives and reacts to the risk of conflict‐related harm.

14 For a stable maximum to exist, we assume the MC curve is steeper than the MB curve: $\frac{\partial MC}{\partial L_{1}}>\frac{\partial MB}{\partial L_{1}}$.

15 While this variable was not framed in terms of the Russia–Ukraine conflict explicitly, disruptions to energy infrastructure were among the most widely discussed consequences of the conflict for neighboring countries, given the region's dependence on Russian gas transiting through Ukraine. We therefore interpret it as partially capturing the essence of conflict‐related disruptions at the individual level. The mere fact that our results hold after including this control suggests that the labor supply response operates through perceived safety risk rather than concerns about energy‐related economic disruption alone.

16 Even though the effects arising from region‐specific shocks that happen to coincide with 2014 remain a possibility, our placebo tests and the Russia‐border falsification exercise in Section 4.3 provide evidence against such confounders.

17
https://www.europeansocialsurvey.org/about‐ess. Last accessed: March 2026.

18 Note that we use the interview years in each ESS wave, rather than the years of the ESS wave, in all our regressions, unless otherwise noted.

19 Not all countries participated in all waves. For example, ESS Wave 3, Edition 3.7, does not include Romania. Data coverage for each wave and edition is available at https://ess.sikt.no/en/. The 11th wave includes 2023 and 2024 as the survey years. Last accessed: 15 January 2025.

20 We note that the observations for Romania cover the period between 2008 and 2009 only (i.e., no post‐2014 data), which may not capture the variation of the labor supply within the country before and after the conflict. Therefore, we removed Romania from our sample.

21 Infrastructural and demographic controls include interrupted energy supply, female gender, age, marital status, born in the same country, citizen of the country, unemployed 3 months plus, time spent on media, children at home, knowledge/skill training, and permanently sick. State controls include the state of economy, health services, and education. Trust controls include trust in political parties, trust in the country's parliament, trust in politicians, and trust in the police.

22 Given that the number of observations in the group that reports no increase in risk aversion is relatively small, we re‐estimate Table 4 using bootstrapped standard errors and find that our results are unlikely to be driven by the smaller sample size of this group. For brevity, we leave the results with the bootstrapped standard errors in Table A9 in the Supporting Information.

23 We note that our exposition in Table 4 does not imply an exhaustive list of channels that could have been at play. Instead, we consider the result in Table 4 as evidence for one potential channel through which conflict uncertainty affects labor choice.

24 We re‐conduct the baseline regressions by including year or ESS round fixed effects in Table A10 in the Supporting Information.

25 As defined by the ESS, the contracted hours exclude paid and unpaid overtime.

26 We use the upper bound of 5.13 for $\text{Working Hours}\;{\text{(log)}}_{i,t}$. This is because there is a maximum of 168 h per week, the log of one plus, which is 5.13.

27 We present the sample distribution for Estonia, Finland, Latvia, and Lithuania in Table A5 in the Supporting Information.

28 In Table A11 in the Supporting Information, we control for changes in regional GDP (gross domestic product), and the results hold. We collect the data of regional GDP at the NUTS‐2 level at https://ec.europa.eu/eurostat/databrowser/explore/all/all_themes. Last accessed: 28 March 2026.

29 Although the 2010 estimates are nontrivial in magnitude, the pre‐2014 pattern is not persistent or monotonic.

30 We note that the interaction coefficients in Table 8 are negative in 2008, and are negative and significant in 2010 for some specifications. One possible interpretation is that regions bordering Ukraine were more significantly affected by the 2008 global financial crisis than those farther away. Nonetheless, the overall lack of significance documented in 2011 and 2013 (one out of six specifications is significant for 2013 at the 10% level) supports our pre‐trend and anticipation assumptions for the DiD specifications. In addition, Table A11 in the Supporting Information shows that our results are robust (i.e., the interaction terms are negative and statistically significant across specifications) even with controls for regional GDP growth included.
