# Supplementary material for: Labor Supply, Risk Aversion, and Conflict Uncertainty
Source: Risk Anal. 2026 Jul 14;46(8):e70308. doi: 10.1111/risa.70308 (PMC13366054; doi:10.1111/risa.70308)
Supplement: Supplementary file 1 — Supporting File: supp‐0001‐SuppMat.pdf [file RISA-46-0-s001.pdf]

Supplemental Appendix Accompanying "Labor Supply, Risk Aversion, and Conflict Uncertainty"

A Derivations of Remark 1

A.1 Under the Gain Domain ($C_1 \geq R_1$)

The First-Order Condition (FOC) for the baseline (gain domain) model is:

$$F(L_1, \theta) = \alpha w_1 (w_1 L_1 - R_1)^{\alpha-1} - (1 - L_1)^{-\sigma} - \delta f(\theta)(K)^{\beta} q'(L_1) = 0 \tag{11}$$

It follows that

$$\frac{dL_1^*}{d\theta} = -\frac{\partial F/\partial \theta}{\partial F/\partial L_1}$$

1. **Numerator ($\partial F/\partial \theta$):**
$$\frac{\partial F}{\partial \theta} = -\delta f'(\theta)(K)^{\beta} q'(L_1) < 0$$
This is the direct effect: increasing conflict risk θ increases the marginal cost of working, as $f'(\theta) > 0$.

2. **Denominator ($\partial F/\partial L_1$):** This is the Second-Order Condition (SOC).
$$\frac{\partial F}{\partial L_1} = \frac{\partial^2 U}{\partial L_1^2} = \underbrace{\alpha(\alpha-1)w_1^2(w_1 L_1 - R_1)^{\alpha-2}}_{<0,\text{ as } \alpha<1} - \underbrace{\sigma(1-L_1)^{-\sigma-1}}_{>0} - \underbrace{\delta f(\theta)(K)^{\beta} q''(L_1)}_{>0 \text{ (if } q''\geq 0)}$$
The SOC is $\frac{\partial F}{\partial L_1} < 0$. This is the standard stability condition for a maximum (the MB curve must cut the MC curve from above). Given $\alpha < 1$ (diminishing sensitivity), the MB slope is negative. The MC slope is positive. Thus, $\frac{\partial F}{\partial L_1} = (\text{negative}) - (\text{positive}) < 0$.

3. **Result:**
$$\frac{dL_1^*}{d\theta} = -\frac{\partial F/\partial \theta}{\partial F/\partial L_1} = -\frac{(-)}{(-)} = -(\text{positive}) < 0$$

This proves that an increase in the conflict parameter θ leads to a decrease in optimal labor L_1^*. □

A.2 Under the Loss Domain ($C_1 < R_1$)

Now, we analyze the loss domain ($C_1 < R_1$). The agent's problem is:

$$\max_{L_1} \quad U = \left[-\lambda (R_1 - w_1 L_1)^{\beta} + \frac{(1-L_1)^{1-\sigma}}{1-\sigma} \right] - \delta f(\theta)(K)^{\beta} q(L_1)$$

The FOC is:

$$G(L_1, \theta) = \lambda \beta w_1 (R_1 - w_1 L_1)^{\beta-1} - (1 - L_1)^{-\sigma} - \delta f(\theta)(K)^{\beta} q'(L_1) = 0$$

Here, we note that the baseline λ is on the MB side, whereas the conflict loss function $f(\theta)$ is on the MC side.

By the implicit function theorem, it follows that

$$\frac{dL_1^*}{d\theta} = -\frac{\partial G/\partial\theta}{\partial G/\partial L_1}. \tag{12}$$

We then consider the numerator and denominator of the previous expression separately.

1. **Numerator ($\partial G/\partial\theta$):**

$$\frac{\partial G}{\partial\theta} = -\delta f'(\theta)(K)^\beta q'(L_1) < 0$$

   This is identical to the gain domain, as $f'(\theta) > 0$. Increasing θ only increases the MC.

2. **Denominator ($\partial G/\partial L_1$):** This is the SOC.

$$\frac{\partial G}{\partial L_1} = \frac{\partial^2 U}{\partial L_1^2} = \underbrace{\lambda\beta(\beta-1)w_1(R_1 - w_1L_1)^{\beta-2}(-w_1)}_{\frac{\partial \text{MB}}{\partial L_1}>0 \text{ (since } \beta<1)} - \underbrace{\left(\sigma(1-L_1)^{-\sigma-1} + \delta f(\theta)(K)^\beta q''(L_1)\right)}_{\frac{\partial \text{MC}}{\partial L_1}>0} \tag{13}$$

   Because $\beta < 1$, the value function is convex in losses, so the MB curve is upward-sloping. For a stable maximum to exist, we must assume the MC curve is steeper than the MB curve: $\frac{\partial \text{MC}}{\partial L_1} > \frac{\partial \text{MB}}{\partial L_1}$. This means the SOC, $\frac{\partial G}{\partial L_1}$, must be negative.

3. **Result:**

$$\frac{dL_1^*}{d\theta} = -\frac{\partial G/\partial\theta}{\partial G/\partial L_1} = -\frac{(-)}{(-)} = -(\text{positive}) < 0$$

Thus, our result is robust and unambiguous in the loss domain. □

We also note that the intertemporal discount factor does not play an independent role in our main comparative static. In particular, the sign of $\frac{\partial L_1^*}{\partial\theta}$ does not depend on the discount factor as it enters the FOC only as a scaling parameter for the conflict-related MC term.

B Related Literature

In an influential paper (Kahneman and Tversky, 1974), co-authored with Amos Tversky, Daniel Kahneman introduced the concepts of cognitive biases and heuristics, mental shortcuts people use to make judgments and decisions. Building on Kahneman and Tversky (1974), Kahneman and Tversky (1979) introduced prospect theory, a psychological theory of decision-making under risk. Relatedly, Tversky and Kahneman (1981) demonstrates that how choices are framed can significantly influence people's decisions.

Tversky and Kahneman (1991) extend prospect theory to riskless choices by showing that people are more sensitive to losses than to gains. For example, the pain of losing $1,000 might only be compensated by the pleasure of earning a greater amount. More recently, Kahneman (2003) and Kahneman (2011) advocate for bounded rationality by arguing that traditional economic models with rational expectations fail to explain many real-world behaviors.

Applications of Kahneman's Theories Slovic (1987) provides a comprehensive overview of risk perception, drawing heavily on Kahneman and Tversky's research on judgment and decision-making under uncertainty Kahneman and Tversky (1974). It highlights the role of affect, heuristics, and social factors in shaping risk perceptions. Posner (1998) considers the intersection of law and economics, focusing on the concept of rational choice and its implications for legal decision-making. Specifically, Posner (1998) examines how individuals make choices, often deviating from the traditional economic model of rational decision-making. It examines the impact of cognitive biases, emotions, and social factors on these choices and how such deviations can influence legal outcomes. Slovic et al. (2004) explore the interplay between analytical reasoning and emotional responses in shaping our perception and understanding of risk. The paper argues that while analytical reasoning plays a crucial role in assessing risk, emotional responses, or "affect," often significantly influence our decisions.

Siegrist and Gutscher (2006) examine how people perceive flood risk and how their perceptions correlate with expert-assessed risk levels. Sjöberg (2000) explores various theories explaining risk perception and finds that while technical risk estimates can sometimes influence perception, they often fall short.

Papers Related to Health, Safety, and Environmental Risks Currie et al. (2015) examine the impact of environmental health risks on housing values. The paper uses data on the opening and closing of toxic plants in the United States to analyze how these events affect property values in surrounding neighborhoods and provides evidence that environmental health risks have a substantial negative impact on housing values. Keeler (1994) finds that lowering speed limits doesn't necessarily reduce fatalities, which is likely attributable to drivers compensating by driving faster or speeding. In a related contribution, Ryan (2012) studies the welfare costs of environmental regulations on the Portland cement industry. Their main finding is that static analysis underestimates costs for consumers and can even yield incorrect estimates for incumbent firms.

C Description of Variables

Table Appendix.1: Survey Questions For Variables Used In Analyses

Variables	Code	Questions	Value
Working Choice	pdwrk	Which of these descriptions applies to what you have been doing for the last 7 days? In paid work (or away temporarily) (employee, self-employed, working for your family business) (Coded: 1=Marked, and 0=Not Marked)	Binary
Working Hours	wkhct	What are/were the total 'basic' or contracted hours each week (in your main job), excluding any paid and unpaid overtime?	Numeric
Need to avoid unsafe areas	impsafe	Please listen to each description and tell me how much each person is or is not like you. It is important to her/him to live in secure surroundings. She/he avoids anything that might endanger her/his safety. (Coded: 0=Not like me at all, or Not like me, or A little like me, or Somewhat like me, and 1=Like me or Very much like me)	Binary
Interrupted Energy Supply	wrtcfl & wrtratc	How worried are you that energy supplies could be interrupted by technical failures? (1 = Not at all worried, 2 = Not very worried, 3 = Somewhat worried, 4 = Very worried, 5 = Extremely worried) & How worried are you that energy supplies could be interrupted by terrorist attacks? (1 = Not at all worried, 2 = Not very worried, 3 = Somewhat worried, 4 = Very worried, 5 = Extremely worried) (Coded: 1 = the answers above 3, 0 = otherwise)	Binary
Female Gender	gndr	Gender. (Coded: 1=Female, 0=Male)	Binary
Age	yrbrn	Year of birth. (Age = year of survey minus year of birth).	Numeric
Marital status	maritalb	Legal marital status. (Coded: 1=Legally married or in a legally registered civil union, 0=None of these (never married or never in a legally registered civil union) or legally separated or legally divorced/Civil union dissolved or widowed/Civil partner died or not disclosed)	Binary
Born In The Same Country	brncntr	Were you born in [country]? (Coded: 1=Yes, 0=No)	Binary
Citizen Of The Country	ctzcntr	Are you a citizen of [country]? (Coded: 1=Yes, 0=No)	Binary
Unemployed 3 Months Plus	uemp3m	Have you ever been unemployed and seeking work for more than 3 months? (Coded: 1=Yes, 0=No)	Binary
Time Spent On Media	nwsptot & rdtot&tvtot	On an average weekday, how much time, in total, do you spend reading the newspapers? On an average weekday, how much time, in total, do you spend listening to the radio? On an average weekday, how much time, in total, do you spend watching television? (Coded: 1=More than 2.5 hours, 0=Equal or Less than 2.5 hours)	Binary
Children At Home	chldhm	Children living at home or not (Coded: 0=No, 1=Yes)	Binary
Knowledge/Skills Training	atncrse	During the last twelve months, have you taken any course or attended any lecture or conference to improve your knowledge or skills for work? (Coded: 1=Yes, 0=No)	Binary
Permanently Sick	dsbld	Are you permanently sick or disabled? (Coded: 1=Yes, 0=No)	Binary
State Of Economy	stfeco	On the whole, how satisfied are you with the present state of the economy in [country] (On a scale of 0-10, from 0=Extremely dissatisfied to 10=Extremely satisfied)	Category
State Of Health Services	stfhlth	On the whole, how satisfied are you with the health services in [country]? (On a scale of 0-10, from 0=Extremely dissatisfied to 10=Extremely satisfied)	Category
State Of Education	stfedu	Please say what you think overall about the state of education in [country] nowadays? (On a scale of 0-10, from 0=Extremely bad to 10=Extremely good)	Category
Trust In Political Parties	trstprt	On a scale of 0-10 how much do you personally trust each of the following institutions? The political parties? (On a scale of 0-10, from 0=No trust at all to 10=Complete trust)	Category
Trust In Country's Parliament	trstprl	On a scale of 0-10 how much do you personally trust each of the following institutions? [country]'s parliament? (On a scale of 0-10, from 0=No trust at all to 10=Complete trust)	Category
Trust In Politicians	trstplt	On a scale of 0-10, how much do you personally trust each of the following institutions? politicians? (On a scale of 0-10, from 0=No trust at all to 10=Complete trust)	Category
Trust in the police	trstplc	Please tell me on a scale of 0-10 how much you personally trust the police. (On a scale of 0-10, from 0=No trust at all to 10=Complete trust)	Category

Immigrants Making the Country Better	imwbcnt	Is [country] made a worse or a better place to live by people coming to live here from other countries? (On a scale of 0-10, from 0=least worst at all to 10=worst)	Category
Breathing Problem	hltphbp & hltprbp	Health problems, hampered, last 12 month: breathing problems & Health problems, last 12 months: breathing problems (Coded: 1=Yes, 0=No)	Binary
Noise Problem	paccnois & hltphsh & hltprsh	Problems with accomodation: noise & Health problems, hampered, last 12 month: severe headaches & Health problems, last 12 months: severe headaches (Coded: 1=Yes, 0=No)	Binary
Local Engagement	atnoact & rlgovw & rlgoptp & sclcptp	In the past 12 months, how often did you help with or attend activities organised in your local area? (Coded: 1=the scales below 5, 0=the scales above or equal 5) & Religious/church organisation, last 12 months: voluntary work & Religious/church organisation, last 12 months: participated & Social club etc., last 12 months: participated (Coded: 1=Yes, 0=No)	Binary

Table Appendix.2: Observations in Regions bordering Ukraine

Panel A: Regional Identifier Pre-2010			
Country	Regions near the Ukrainian border	Regional Identifier	Obs.
Poland	Lubelskie	Region Name, PL31 or PL81	452
Poland	Podkarpackie	Region Name, PL32 or PL82	431
Poland	Podlaskie	Region Name, PL34 or PL84	237
Hungary	North Region	Region Name, including HU311	796
Hungary	North Plain	Region Name, including HU323	962
Slovakia	Prešovský kraj	Region Name, SK041	728
Slovakia	Košický kraj	Region Name, SK042	734

Panel B: Regional Identifier Post-2010			
Country	Regions near the Ukrainian border	Regional Identifier	Obs.
Poland	Lubelskie	PL31 or PL81	688
Poland	Podkarpackie	PL32 or PL82	745
Poland	Podlaskie	PL34 or PL84	384
Hungary	Borsod-Abaúj-Zemplén	HU311	1,011
Hungary	Szabolcs-Szatmár-Bereg	HU323	838
Slovakia	Košický kraj	SK042	916
Slovakia	Prešovský kraj	SK041	1,232

Figure Appendix.1: Estimates using Randomized Timing, using the years before the conflict timing

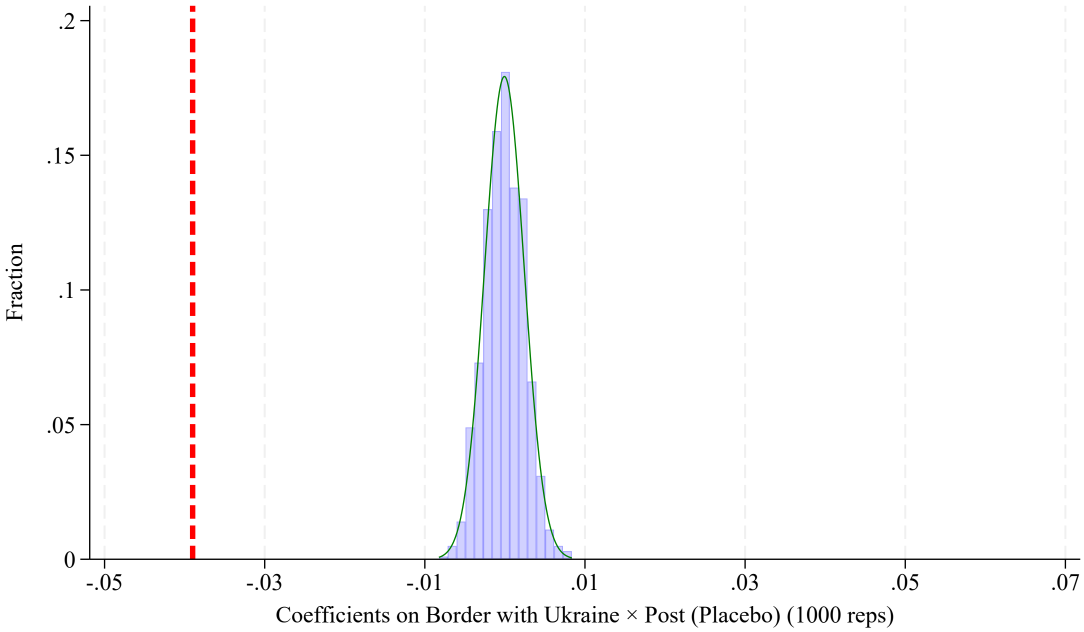

Note: This figure displays a placebo test using a placebo measurement *Post (Placebo)* instead of the actual *Post 2014*. We use the years before the conflict timing (i.e., from 2002 to 2013) and repeat the randomization 1,000 replications and report the distribution of the estimated coefficients on Border with Ukraine × Post (Placebo). The dependent variable is Working choice. The true estimate using actual *Border with Ukraine* × Post 2014 from our baseline result is overlaid as a red vertical line in the figure.

Figure Appendix.2: Coefficients of The Interaction Term Before and After 2014

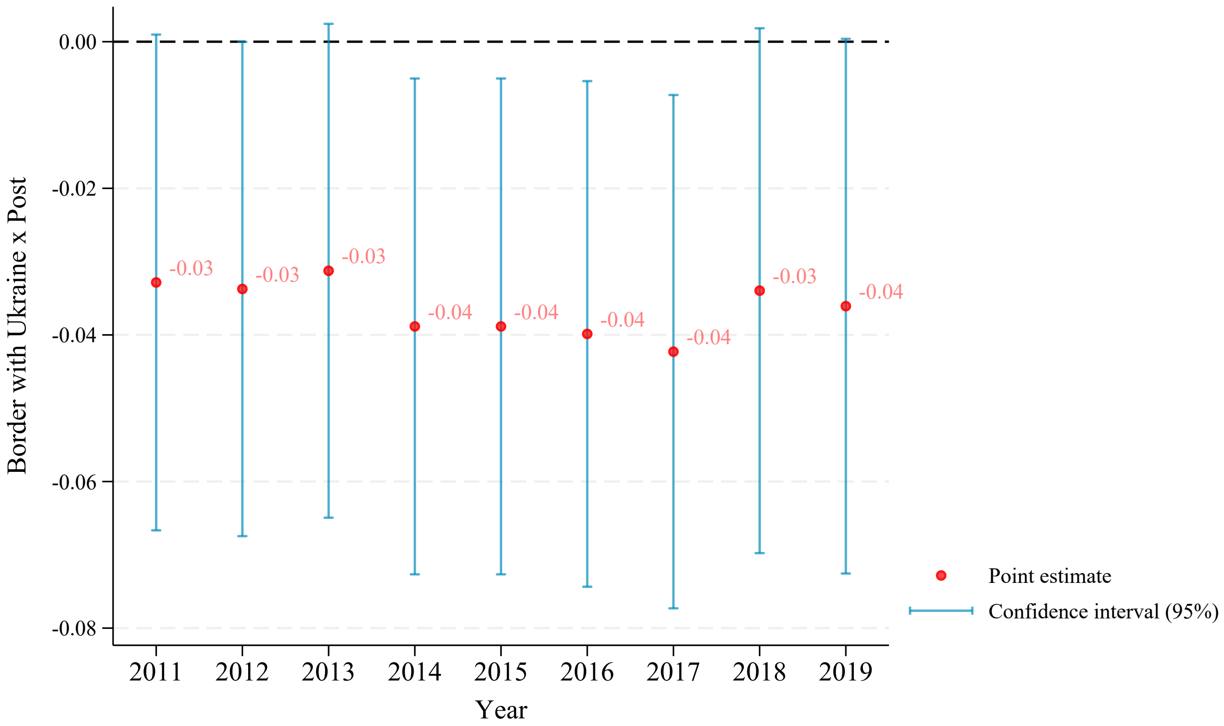

Note: This figure presents year-specific estimates of the effect of the Russia–Ukraine conflict on the working choice of individuals in regions bordering Ukraine over the period 2011–2019. Each dot represents the estimated coefficient of the interaction between year-specific Post indicators (2011–2019) and Border with Ukraine, while the vertical bars indicate 95% confidence intervals. The dashed horizontal line denotes zero. The coefficients and standard errors are computed using the same specification and estimation procedure as in the main analysis.

Discussion: This figure presents coefficients from a series of separate regressions, each using a different year as the cutoff for the Post indicator. Specifically, for each year $t \in \{2011, \ldots, 2019\}$, we re-estimate the baseline specification with Post_t replacing Post_{2014}, and plot the resulting coefficient on Border with $\text{Ukraine}_i \times \text{Post}_t$. As a result, each plotted coefficient is therefore a standalone difference-in-differences estimate for that particular cutoff year. When $t = 2014$, the coefficient in the figure corresponds directly to our baseline estimate. We note that Figure Appendix.2 and Table 8 use different identifying variation and, as a result, the coefficients are not directly comparable. In Figure Appendix.2, each plotted point comes from a separate regression in which the Post indicator is redefined using a different cutoff year. Here, each coefficient is therefore a standalone difference in differences estimate for that particular cutoff. In Table 8, by contrast, we interact the border indicator with individual ESS wave (or interview year) dummies against a 2012 baseline within a single regression, while pooling the post 2014 observations. The former captures the cumulative treatment effect at each hypothetical cutoff, whereas the latter isolates wave specific deviations from the omitted baseline year.

Table Appendix.3: Descriptive statistics: Difference-in-means between two groups

Panel A: Full sample

	Without Border with Ukraine		With Border with Ukraine		Pairwise t-test		
	Obs.	Mean	Obs.	Mean	Obs.	Mean Difference	Std. Error
Working Choice	40,315	0.512	10,125	0.462	50,440	0.050***	0.006
Working Hours	33,545	54.336	8,169	49.426	41,714	4.910***	0.988
Working Hours (Log)	33,545	3.765	8,169	3.736	41,714	0.029***	0.006
Need To Avoid Unsafe Areas	37,693	0.916	9,632	0.935	47,325	-0.019***	0.003
Interrupted Energy Supply	40,315	0.951	10,125	0.964	50,440	-0.013***	0.002
Female Gender	40,258	0.553	10,102	0.541	50,360	0.012**	0.006
Age	40,058	47.802	10,062	47.848	50,120	-0.046	0.206
Marital Status	40,315	0.316	10,125	0.295	50,440	0.021***	0.005
Born In The Same Country	40,282	0.984	10,116	0.983	50,398	0.000	0.001
Citizen Of The Country	40,277	0.996	10,119	0.996	50,396	0.000	0.001
Unemployed Past 3 Months	40,315	0.260	10,125	0.297	50,440	-0.038***	0.005
Time Spent On Media	40,315	0.799	10,125	0.768	50,440	0.031***	0.005
Children At Home	40,180	0.412	10,090	0.435	50,270	-0.024***	0.005
Knowledge/Skills Training	40,315	0.187	10,125	0.187	50,440	-0.000	0.004
Permanently Sick	40,315	0.027	10,125	0.038	50,440	-0.011***	0.002
State Of Economy	38,979	4.028	9,777	3.802	48,756	0.226***	0.027
State Of Health Services	39,439	3.733	9,667	3.996	49,106	-0.263***	0.028
State Of Education	37,013	5.016	9,118	5.143	46,131	-0.128***	0.028
Trust In Political Parties	36,369	2.941	9,034	2.895	45,403	0.047*	0.028
Trust In Country's Parliament	39,285	3.630	9,845	3.607	49,130	0.023	0.029
Trust In Politicians	39,398	2.956	9,878	2.889	49,276	0.067**	0.027
Trust In The Police	39,623	5.206	9,940	5.187	49,563	0.019	0.029

Panel B: Pre-2014

	Without Border with Ukraine		With Border with Ukraine		Pairwise t-test		
	Obs.	Mean	Obs.	Mean	Obs.	Mean Difference	Std. Error
Working Choice	22,999	0.485	6,396	0.444	29,395	0.041***	0.007
Working Hours	18,942	40.735	5,117	40.693	24,059	0.042	0.147
Working Hours (Log)	18,942	3.699	5,117	3.693	24,059	0.005	0.005
Need To Avoid Unsafe Areas	22,496	0.930	6,259	0.938	28,755	-0.008**	0.004
Interrupted Energy Supply	22,999	1.000	6,396	1.000	29,395	0.000	0.000
Female Gender	22,942	0.546	6,373	0.543	29,315	0.003	0.007
Age	22,881	46.191	6,369	46.348	29,250	-0.157	0.261
Marital Status	22,999	0.199	6,396	0.180	29,395	0.019***	0.006
Born In The Same Country	22,981	0.980	6,390	0.984	29,371	-0.004**	0.002
Citizen Of The Country	22,982	0.998	6,394	0.998	29,376	-0.000	0.001
Unemployed Past 3 Months	22,999	0.292	6,396	0.323	29,395	-0.031***	0.006
Time Spent On Media	22,999	0.648	6,396	0.633	29,395	0.015**	0.007
Children At Home	22,864	0.450	6,361	0.479	29,225	-0.029***	0.007
Knowledge/Skills Training	22,999	0.213	6,396	0.213	29,395	0.000	0.006
Permanently Sick	22,999	0.033	6,396	0.045	29,395	-0.012***	0.003
State Of Economy	22,183	3.658	6,170	3.456	28,353	0.202***	0.032
State Of Health Services	22,310	3.745	5,977	3.949	28,287	-0.204***	0.036
State Of Education	21,005	5.107	5,593	5.210	26,598	-0.103***	0.035
Trust In Political Parties	19,389	2.705	5,399	2.733	24,788	-0.028	0.034
Trust In Country's Parliament	22,314	3.445	6,220	3.491	28,534	-0.046	0.035
Trust In Politicians	22,389	2.759	6,224	2.779	28,613	-0.021	0.032
Trust In The Police	22,514	4.875	6,266	4.899	28,780	-0.023	0.036

Notes: This table presents the mean differences in our variables of interest between the two groups: those with Border with Ukraine and those without in Panel A (Full sample) and Panel B (Pre-2014).

Table Appendix.4: Correlations

	(1)	(2)	(3)	(4)	(5)	(6)	(7)	(8)	(9)	(10)	(11)	(12)	(13)	(14)	(15)	(16)	(17)	(18)	(19)	(20)	(21)	(22)	(23)
(1) Working Choice	1.00																						
(2) Working Hours (Log)	0.02***	1.00																					
(3) Need To Avoid Unsafe Areas	-0.03***	-0.02***	1.00																				
(4) Border with Ukraine	-0.04***	-0.02***	0.03***	1.00																			
(5) Post 2014	0.05***	0.11***	-0.06***	-0.03***	1.00																		
(6) Interrupted Energy Supply	-0.03***	0.03***	0.01	0.02***	-0.26***	1.00																	
(7) Female Gender	-0.11***	-0.07***	0.05***	-0.02***	0.01**	0.01**	1.00																
(8) Age	-0.50***	0.05***	0.04***	0.01**	0.11***	0.00	0.05***	1.00															
(9) Marital Status	0.05***	0.05***	-0.01	-0.02***	0.32***	-0.07***	-0.03***	0.13***	1.00														
(10) Born In The Same Country	0.04***	0.01	-0.01**	-0.01*	0.03***	-0.01*	-0.01*	-0.05***	0.01**	1.00													
(11) Citizen Of The Country	-0.01	-0.04***	0.03***	0.00	-0.04***	-0.01	-0.00	-0.01	-0.02***	0.15***	1.00												
(12) Unemployed Past 3 Months	0.03***	-0.03***	0.00	0.04***	-0.12***	0.02***	-0.01**	-0.22***	-0.07***	0.01	0.00	1.00											
(13) Time Spent On Media	-0.05***	0.07***	-0.01*	-0.02***	0.42***	-0.11***	0.02***	0.11***	0.23***	0.00	-0.02***	-0.04***	1.00										
(14) Children At Home	0.03***	-0.01	0.02***	0.02***	-0.11***	0.02***	0.03***	0.06***	0.12***	0.00	-0.01	0.07***	-0.09***	1.00									
(15) Knowledge/Skills Training	0.27***	-0.05***	-0.01***	0.01	-0.11***	0.02***	-0.02***	-0.27***	-0.07***	0.00	0.01**	0.01***	-0.11***	0.01**	1.00								
(16) Permanently Sick	-0.16***	-0.01**	0.01*	0.03***	-0.05***	0.01**	-0.01*	0.06***	-0.04***	-0.01	0.00	0.03***	-0.00	-0.01**	-0.05***	1.00							
(17) State Of Economy	0.05***	0.02***	-0.04***	-0.04***	0.19***	-0.09***	-0.01	0.02***	0.10***	-0.01	-0.01**	-0.12***	0.08***	-0.03***	0.02***	-0.06***	1.00						
(18) State Of Health Services	-0.03***	0.02***	-0.02***	0.04***	0.03***	-0.02***	0.01**	0.02***	0.01***	-0.02***	-0.03***	-0.05***	0.02***	-0.03***	-0.05***	-0.01**	0.41***	1.00					
(19) State Of Education	-0.02***	0.01**	0.01	0.02***	-0.01**	-0.03***	0.02***	-0.01**	0.01**	-0.02***	-0.01	-0.01**	-0.00	0.01	-0.03***	-0.02***	0.40***	0.51***	1.00				
(20) Trust In Political Parties	-0.02***	0.03***	-0.02***	-0.02***	0.14***	-0.01*	0.04***	0.09***	0.07***	-0.02***	-0.02***	-0.12***	0.07***	-0.05***	-0.05***	-0.02***	0.42***	0.35***	0.29***	1.00			
(21) Trust In Country's Parliament	-0.00	0.03***	-0.01	-0.01**	0.13***	-0.03***	0.03***	0.08***	0.08***	-0.02***	-0.02***	-0.10***	0.05***	-0.04***	-0.01**	-0.02***	0.45***	0.33***	0.30***	0.70***	1.00		
(22) Trust In Politicians	-0.02***	0.03***	-0.02***	-0.03***	0.15***	-0.02***	0.05***	0.10***	0.07***	-0.02***	-0.03***	-0.12***	0.07***	-0.04***	-0.05***	-0.02***	0.44***	0.36***	0.31***	0.87***	0.74***	1.00	
(23) Trust In The Police	0.01	0.02***	0.01*	-0.01	0.17***	-0.08***	0.04***	0.06***	0.08***	-0.00	-0.01**	-0.08***	0.07***	-0.03***	-0.01***	-0.02***	0.34***	0.28***	0.28***	0.45***	0.49***	0.49***	1.00

Notes: This table presents correlations between the variables. Details of variable definitions and construction are presented in Table Appendix.1. Significance levels are denoted as follows: * for $p < 0.10$, ** for $p < 0.05$, and *** for $p < 0.01$.

Table Appendix.5: Sample Distribution for Countries bordering Russia only

	Estonia		Finland		Latvia		Lithuania	
	Obs.	%	Obs.	%	Obs.	%	Obs.	%
2002			2,000	9.487				
2004	1,400	8.563	2,022	9.591				
2005	589	3.602						
2006	641	3.920	1,896	8.993				
2007	876	5.358						
2008	458	2.801	1,929	9.150				
2009	1,203	7.358	266	1.262	1,980	38.984		
2010			1,877	8.903				
2011							1,610	12.902
2012	2,279	13.939	1,883	8.932				
2013	101	0.618	313	1.485			2,011	16.115
2014	2,049	12.532	1,913	9.074				
2015			172	0.816			2,161	17.317
2016	1,806	11.046	1,548	7.343				
2017	212	1.297	375	1.779			2,067	16.564
2018	1,710	10.459	1,499	7.110				
2019	194	1.187	253	1.200	835	16.440	1,704	13.655
2020					1,026	20.201		
2021	1,541	9.425	1,568	7.438			1,599	12.814
2022			8	0.038				
2023			1,516	7.191	462	9.096	1,327	10.634
2024	1,291	7.896	44	0.209	776	15.279		
Total	16,350	100.000	21,082	100.000	5,079	100.000	12,479	100.000

Notes: This table presents the sample distribution across the countries that share borders with Russia only (i.e., Estonia, Finland, Latvia, and Lithuania) across years.

Table Appendix.6: Robustness Tests: Russia Border

	Working Choice		
	(1)	(2)	(3)
Border with Russia × Post 2014	0.003	0.001	-0.001
	(0.015)	(0.016)	(0.017)
Border with Russia	-0.020*	-0.015	-0.015
	(0.011)	(0.011)	(0.013)
Post 2014	0.020***	0.020**	0.023***
	(0.008)	(0.008)	(0.008)
Demographic Controls	Yes	Yes	Yes
State Controls	No	Yes	Yes
Trust Controls	No	No	Yes
Income Sources FE	Yes	Yes	Yes
Occupation FE	Yes	Yes	Yes
Highest Education FE	Yes	Yes	Yes
Country FE	Yes	Yes	Yes
Pseudo R-squared	0.521	0.511	0.522
Observations	56,576	53,160	49,779

Notes: This table presents the results of Probit regressions concerning the impact of increased conflict uncertainty (i.e., due to the Russian Annexation of Crimea 2014) on Individual Working Choice. The dependent variable is Working Choice. This binary variable indicates whether an individual has worked in the last 7 days (i.e., employee, self-employed, or working for a family business). Border with Russia is a dummy variable that indicates whether an individual resides in a locality with a border with Russia. Post 2014 is an indicator function that takes a value of one if the observation is in or after 2014 and zero otherwise. Our interest is in the coefficients of Border with Russia × Post 2014. The regions bordering Russia include Warmińsko-Mazurskie (in Poland), Kirde-Eesti and Lõuna-Eesti (in Estonia), Latgale and Vidzeme (in Latvia), Klaipėdos apskritis and Tauragės apskritis (in Lithuania), and Pohjois-Karjala, Kainuu, and Lappi (in Finland). Our control variables include individual demographics, the state of the economy, the state of health services, the state of education, and trust. More details of control variables are presented in Table Appendix.1. We include income sources, occupation, highest education level, and country fixed effects. Robust standard errors are shown in parentheses. Significance levels are denoted as follows: * for $p < 0.10$, ** for $p < 0.05$, and *** for $p < 0.01$.

Table Appendix.7: Robustness: Working Hours (Log) and Alternative Clustering For Standard Errors

	Working Hours (Log)			
	(1)	(2)	(3)	(4)
Border with Ukraine × Post 2014	-0.026**	-0.026**	-0.026**	-0.026**
	(0.011)	(0.011)	(0.011)	(0.011)
Border with Ukraine	-0.008	-0.008	-0.008	-0.008
	(0.006)	(0.006)	(0.006)	(0.006)
Post 2014	0.097***	0.097***	0.097***	0.097***
	(0.006)	(0.006)	(0.006)	(0.006)
Demographic Controls	Yes	Yes	Yes	Yes
State Controls	Yes	Yes	Yes	Yes
Trust Controls	Yes	Yes	Yes	Yes
Income Sources FE	Yes	Yes	Yes	Yes
Occupation FE	Yes	Yes	Yes	Yes
Highest Education FE	Yes	Yes	Yes	Yes
Country FE	Yes	Yes	Yes	Yes
Pseudo R-squared	0.056	0.056	0.056	0.056
Observations	33,734	33,734	33,734	33,734
Clusters	Robust	ID × Country	ID × Region	ID × ESS Round

Notes: This table presents the results of Tobit regressions concerning the impact of conflict uncertainty on Individual Working Hours. The dependent variable is Working Hours (Log), which is the natural logarithm of one plus the total contracted working hours per week in the main job. From Table 1, the maximum recorded value for working hours is 555. To account for this coding issue, we estimate a Tobit model with an upper bound of 5.13, corresponding to the logarithm of 168 hours, the maximum number of hours available in a week. Column (1) reproduces the baseline Tobit regression, similarly in Table 5. In Column (2), standard errors are clustered at the participant and country levels, while in Column (3) they are clustered at the participant and region levels. Column (4) reports standard errors clustered at the participant and ESS round levels. The standard errors are shown in parentheses. Border with Ukraine is a dummy variable that indicates whether an individual resides in a locality near the Ukrainian border. Post 2014 is an indicator function that takes a value of one if the observation is in or after 2014 and zero otherwise. Our interest is the coefficients of Border with Ukraine × Post 2014. Our control variables include individual demographics, the state of the economy, the state of health services, the state of education, and trust. More details of control variables are presented in Table Appendix.1. We include income sources, occupation, highest education level, and country fixed effects. Significance levels are denoted as follows: * for $p < 0.10$, ** for $p < 0.05$, and *** for $p < 0.01$.

Table Appendix.8: Robustness: Anticipation Effects

	Working Choice		
	(1)	(2)	(3)
Border with Ukraine × Post 2011			0.007
			(0.074)
Post 2011			-0.039
			(0.034)
Border with Ukraine × Post 2012		-0.028	-0.035
		(0.031)	(0.078)
Post 2012		0.005	0.042
		(0.014)	(0.035)
Border with Ukraine × Post 2013	0.128*	0.151**	0.150**
	(0.068)	(0.073)	(0.073)
Post 2013	-0.066**	-0.070**	-0.071**
	(0.031)	(0.033)	(0.033)
Border with Ukraine × Post 2014	-0.164**	-0.163**	-0.163**
	(0.068)	(0.068)	(0.068)
Post 2014	0.148***	0.148***	0.149***
	(0.031)	(0.031)	(0.031)
Border with Ukraine	-0.012	-0.007	-0.007
	(0.012)	(0.013)	(0.013)
Demographic Controls	Yes	Yes	Yes
State Controls	Yes	Yes	Yes
Trust Controls	Yes	Yes	Yes
Income Sources FE	Yes	Yes	Yes
Occupation FE	Yes	Yes	Yes
Highest Education FE	Yes	Yes	Yes
Country FE	Yes	Yes	Yes
Pseudo R-squared	0.494	0.494	0.494
Observations	39,820	39,820	39,820

Notes: This table presents the results of Probit regressions concerning the impact of increased conflict uncertainty (i.e., due to the Russian Annexation of Crimea 2014) on Individual Working Choice. The dependent variable is Working Choice. This binary variable indicates whether an individual has worked in the last 7 days (i.e., employee, self-employed, or working for a family business). Border with Ukraine is a dummy variable that indicates whether an individual resides in a locality with a border with Ukraine. Post 2014 is an indicator function that takes a value of one if the observation is in or after 2014 and zero otherwise. Our interest is on the coefficients of Border with Ukraine × Post 2014. We also include Post 2011 which is an indicator function that takes a value of one if the observation is in or after 2011 and zero otherwise. Post 2012 is an indicator function that takes a value of one if the observation is in or after 2012 and zero otherwise. Post 2013 is an indicator function that takes a value of one if the observation is in or after 2013 and zero otherwise. We also obtain the interaction terms Border with Ukraine × Post 2011, Border with Ukraine × Post 2012, and Border with Ukraine × Post 2013. Our control variables include individual demographics, the state of the economy, the state of health services, the state of education, and trust. More details of control variables are presented in Table Appendix.1. We include income sources, occupation, highest education level, and country fixed effects. Robust standard errors are shown in parentheses. Significance levels are denoted as follows: * for $p < 0.10$, ** for $p < 0.05$, and *** for $p < 0.01$.

Table Appendix.9: Robustness: Conflict Uncertainty and Individual Working Choice Under the Perspective of Safety - Using Bootstrap for Standard Errors

	Working Choice (Need To Avoid Unsafe Areas=1)			Working Choice (Need To Avoid Unsafe Areas=0)		
	(1)	(2)	(3)	(4)	(5)	(6)
Border with Ukraine × Post 2014	-0.045***	-0.052***	-0.051***	0.016	0.021	0.037
	(0.017)	(0.018)	(0.018)	(0.060)	(0.062)	(0.069)
Border with Ukraine	-0.010	-0.007	-0.008	-0.011	-0.035	-0.044
	(0.010)	(0.011)	(0.012)	(0.040)	(0.043)	(0.048)
Post 2014	0.089***	0.091***	0.077***	0.065**	0.075**	0.049
	(0.009)	(0.010)	(0.010)	(0.030)	(0.031)	(0.033)
Demographic Controls	Yes	Yes	Yes	Yes	Yes	Yes
State Controls	Yes	Yes	Yes	Yes	Yes	Yes
Trust Controls	Yes	Yes	Yes	Yes	Yes	Yes
Income Sources FE	Yes	Yes	Yes	Yes	Yes	Yes
Occupation FE	Yes	Yes	Yes	Yes	Yes	Yes
Highest Education FE	Yes	Yes	Yes	Yes	Yes	Yes
Country FE	Yes	Yes	Yes	Yes	Yes	Yes
Pseudo R-squared	0.497	0.486	0.501	0.460	0.449	0.466
Observations	43,141	38,325	34,160	3,748	3,355	3,044

Notes: This table presents the results of Probit regressions concerning the impact of increased conflict uncertainty on Individual Working Choice, across different degrees of risk aversion. The dependent variable is Working Choice. This binary variable indicates whether an individual has been working in the past 7 days (i.e., employed, self-employed, or working in a family business). Border with Ukraine is a dummy variable that indicates whether an individual resides in a locality with a border with Ukraine. Post 2014 is an indicator function that takes a value of one if the observation is in or after 2014 and zero otherwise. Our interest is in the coefficients of Border with Ukraine × Post 2014. Columns (1)-(3) use a subsample where the value of Need To Avoid Unsafe Areas is one, meaning the individuals are risk averse (to avoid unsafe areas to live). In contrast, Columns (4)-(6) use a subsample where the value of Need To Avoid Unsafe Areas is zero, meaning the individuals are not risk-averse. Our control variables include individual demographics, economic conditions, health service conditions, educational conditions, and trust. More details of control variables are presented in Table Appendix.1. We include income sources, occupation, highest education level, and country fixed effects. We use bootstrapped standard errors and show them in parentheses. Significance levels are denoted as follows: * for $p < 0.10$, ** for $p < 0.05$, and *** for $p < 0.01$.

Table Appendix.10: Robustness: Including Year or ESS round Fixed Effects

	Working Choice		Need To Avoid Unsafe Areas	
	(1)	(2)	(3)	(4)
Border with Ukraine × Post 2014	-0.042**	-0.040**	0.027***	0.027***
	(0.017)	(0.017)	(0.007)	(0.007)
Border with Ukraine	-0.009	-0.010	0.004	0.005
	(0.012)	(0.012)	(0.005)	(0.005)
Post 2014	-0.017	0.100***	-0.045***	-0.045***
	(0.040)	(0.018)	(0.015)	(0.007)
Demographic Controls	Yes	Yes	Yes	Yes
State Controls	Yes	Yes	Yes	Yes
Trust Controls	Yes	Yes	Yes	Yes
Income Sources FE	Yes	Yes	Yes	Yes
Occupation FE	Yes	Yes	Yes	Yes
Highest Education FE	Yes	Yes	Yes	Yes
Country FE	Yes	Yes	Yes	Yes
Year FE	Yes	No	Yes	No
ESS round FE	No	Yes	No	Yes
Pseudo R-squared	0.496	0.495	0.031	0.030
Observations	39,820	39,820	37,204	37,204

Notes: This table presents the results of Probit regressions concerning the impact of increased conflict uncertainty on Individual Working Choice (Working Choice in Columns 1-2) and safety choice (Need To Avoid Unsafe Areas in Columns 3-4). Border with Ukraine is a dummy variable that indicates whether an individual resides in a locality with a border with Ukraine. Post 2014 is an indicator function that takes a value of one if the observation is in or after 2014 and zero otherwise. Our interest is in the coefficients of Border with Ukraine × Post 2014. Our control variables include individual demographics, economic conditions, health service conditions, educational conditions, and trust. More details of control variables are presented in Table Appendix.1. We include income sources, occupation, highest education level, and country fixed effects. We also include year fixed effects in Columns (1) and (3), while ESS round fixed effects are included in Columns (2) and (4). We use robust standard errors and show them in parentheses. Significance levels are denoted as follows: * for $p < 0.10$, ** for $p < 0.05$, and *** for $p < 0.01$.

Table Appendix.11: Robustness: Controlling for Changes in Regional GDP (Gross Domestic Product)

	Working Choice			
	(1)	(2)	(3)	(4)
Border with Ukraine × Post 2014	-0.029*	-0.039*	-0.053***	-0.046**
	(0.017)	(0.020)	(0.020)	(0.020)
Border with Ukraine	-0.000	-0.004	-0.001	-0.003
	(0.011)	(0.014)	(0.014)	(0.014)
Post 2014	0.076***	0.082***	-0.042	0.091***
	(0.008)	(0.010)	(0.041)	(0.019)
Changes in Regional GDP	0.059	0.160***	0.414***	0.203***
	(0.045)	(0.056)	(0.136)	(0.065)
Demographic Controls	No	Yes	Yes	Yes
State Controls	No	Yes	Yes	Yes
Trust Controls	No	Yes	Yes	Yes
Income Sources FE	Yes	Yes	Yes	Yes
Occupation FE	Yes	Yes	Yes	Yes
Highest Education FE	Yes	Yes	Yes	Yes
Country FE	Yes	Yes	Yes	Yes
Year FE	No	No	Yes	No
ESS round FE	No	No	No	Yes
Pseudo R-squared	0.454	0.493	0.495	0.494
Observations	40,632	31,999	31,999	31,999

Notes: This table presents the results of Probit regressions concerning the impact of increased conflict uncertainty on Individual Working Choice (Working Choice). Border with Ukraine is a dummy variable that indicates whether an individual resides in a locality with a border with Ukraine. Post 2014 is an indicator function that takes a value of one if the observation is in or after 2014 and zero otherwise. Our interest is in the coefficients of Border with Ukraine × Post 2014. Changes in Regional GDP is the regional GDP growth (Gross Domestic Product growth). Our control variables include individual demographics, economic conditions, health service conditions, educational conditions, and trust. More details of control variables are presented in Table Appendix.1. We include income sources, occupation, highest education level, and country fixed effects. We also include year fixed effects in Column (3) and ESS round fixed effects in Column (4). We use robust standard errors and show them in parentheses. Significance levels are denoted as follows: * for $p < 0.10$, ** for $p < 0.05$, and *** for $p < 0.01$.
